# Consensus‐Based Recommendations on Pulp Therapies in Primary and Permanent Teeth: IAPD Porto Forum

**DOI:** 10.1111/ipd.70068

**Published:** 2026-02-02

**Authors:** Vineet Dhar, Ikhlas El‐Karim, James A. Coll, Ashraf F. Fouad, Anne C. O'Connell, Saeed Asgary, Lars Bjørndal, Zafer C. Cehreli, Yasmi O. Crystal, Manikandan Ekambaram, Brian D. Hodgson, Nicola P. Innes, Jonas Almeida Rodrigues, Nessrin A. Taha, Nitesh Tewari, Tugba Turk

**Affiliations:** ^1^ Department of Orthodontics and Pediatric Dentistry University of Maryland, School of Dentistry Baltimore Maryland USA; ^2^ School of Medicine Dentistry and Biomedical Sciences Queen's University Belfast Belfast UK; ^3^ The Department of Endodontics School of Dentistry, University of Alabama Birmingham Alabama USA; ^4^ Division of Public and Child Dental Health Dublin Dental University Hospital, Trinity College Dublin Ireland; ^5^ Iranian Center for Endodontic Research, Research Institute of Dental Sciences Shahid Beheshti University of Medical Sciences Tehran Iran; ^6^ Cariology and Endodontics, Section of Clinical Oral Microbiology, Department of Odontology, Faculty of Health and Medical Sciences University of Copenhagen Copenhagen Denmark; ^7^ Department of Pediatric Dentistry, Faculty of Dentistry Hacettepe University Ankara Turkey; ^8^ Department of Pediatric Dentistry New York University New York New York USA; ^9^ Department of Oral Sciences, Faculty of Dentistry University of Otago Dunedin New Zealand; ^10^ Marquette University School of Dentistry Milwaukee USA; ^11^ School of Dentistry, Professor and Head of the School of Dentistry, College of Biomedical and Life Sciences Cardiff University Cardiff UK; ^12^ Universidade Federal do Rio Grande do Sul (UFRGS) Porto Alegre Brazil; ^13^ Department of Conservative Dentistry, Faculty of Dentistry Jordan University of Science and Technology Irbid Jordan; ^14^ Pediatric and Preventive Dentistry, Centre for Dental Education and Research, All India Institute of Medical Sciences New Delhi India; ^15^ Ege University, School of Dentistry, Department of Endodontics Izmir Turkey

**Keywords:** endodontics, pulp biology/basic sciences, traumatic injuries

## Abstract

**Background:**

Recent understanding of pulp biology has shifted treatment paradigms toward preservation‐based approaches. Traditional diagnostic terminology and treatment protocols require updating to align with current evidence supporting the pulp's healing capacity.

**Aim:**

Sixteen international specialists in pediatric dentistry and endodontics convened at the 3rd IAPD Summit in Porto, Portugal (November 2024) to develop consensus‐based recommendations on pulp therapies in primary and permanent teeth.

**Methods:**

Following a structured three‐phase approach, experts conducted systematic literature reviews and participated in Delphi surveys using a 7‐point Likert scale. Recommendations achieving > 70% consensus were categorized as strong (based on RCTs/systematic reviews), conditional (observational studies), or consensus‐based statements (expert opinion).

**Results:**

Thirty‐eight evidence‐based recommendations were developed across four key areas: pulp inflammation and diagnosis, caries excavation, management of pulpitis in primary and permanent teeth, and traumatic dental injuries. Key findings emphasized selective caries removal over complete excavation, calcium silicate cements as preferred materials for vital pulp therapy, and conservative approaches for managing irreversible pulpitis. Decision trees were created to support clinical implementation.

**Conclusions:**

These consensus recommendations provide evidence‐based guidance for managing pulpal diseases using minimally invasive, biologically driven tiered approaches that prioritize pulp preservation in both primary and permanent teeth.

## Why Is This Paper Important to Pediatric Dentists?

1. Provides protocols for diagnosing and using a tiered approach, prioritizing conservative methods as the first‐line treatment for managing primary and young permanent teeth needing pulp therapies due to caries or trauma.
2. The 38 recommendations and decision trees offer clear, evidence‐based guidelines for managing deep caries, extremely deep caries, and trauma.
3. The consensus identifies calcium silicate cements as the preferred choice over traditional materials and advocates for selective caries removal rather than complete excavation in teeth with deep caries.

## 1 Introduction

Managing pulpal and apical diseases in primary and permanent teeth is a critical aspect of dental care. Recent advances have transformed our understanding of pulp biology. Current research recognizes pulp's healing capacity and defensive mechanisms, which have shifted treatment paradigms for both primary and permanent dentitions [1, 2]. Traditional perspectives viewed pulpal inflammation as a largely progressive condition often culminating in pulp death. This led to treatment approaches that favored complete pulp removal, even when pulp was partially involved.

Contemporary research reveals that dental pulp possesses remarkable defensive and reparative capabilities, which may be leveraged through minimally invasive interventions [3, 4, 5]. The inflammatory process, once viewed primarily as destructive, is now recognized as a complex biological response that can facilitate healing and repair when appropriately managed [4]. The pulp responds to injury, whether from caries or trauma, through complex cellular and molecular mechanisms.

These mechanisms include recruiting immune cells, releasing inflammatory mediators, and activating dental pulp stem cells (DPSCs). In slowly progressing carious lesions, bacterial products diffuse through dentinal tubules. This triggers a controlled inflammatory response associated with increased odontoblast activity and reactionary dentin formation [5]. More severe injury causes odontoblast cell death. This results in an inflammatory response that, if properly managed, can lead to DPSC activation and differentiation into odontoblast‐like cells, producing reparative dentin [6].

The traditional diagnostic terminology (reversible/irreversible pulpitis) is becoming increasingly inadequate [7], with alternative classifications proposed [8, 9, 10] and consensus efforts underway to align terminology with current evidence. Additionally, there is a lack of consensus on the pulpal diagnostic terminology used by pediatric dentists and endodontists [10, 11, 12]. Clinical evidence suggests that the Wolters classification system may offer a prognostic advantage over traditional American Association of Endodontics (AAE) terminology, especially within the subdivisions of irreversible pulpitis [13].

Accurate diagnosis of pulp status remains a significant challenge. Clinical symptoms and traditional testing methods may not reliably reflect the histological status of the pulp. The limitations of current diagnostic tools for pulpitis, particularly in assessing the inflammatory status of the pulp, have prompted investigation into biomarkers for pulpal diagnosis and prognosis [14, 15]. While the evidence is emerging, no established thresholds yet support the routine use of biomarkers in clinical practice [16].

Recent evidence has challenged traditional treatment paradigms, particularly regarding the management of deep caries, pulp exposures, and pulp injury following dental traumatic injuries. The concept of “irreversible pulpitis” is being reconsidered as evidence emerges that inflamed pulp tissue, once considered irreversibly damaged, may have healing potential under appropriate conditions [17]. Consequently, there is a growing emphasis on pulp preservation using minimally invasive pulp therapies such as partial pulpotomy (PP), which consider pulpal inflammation as existing on a continuum and aim to preserve pulp vitality and function [18].

The development of bioactive materials, particularly calcium silicate‐based cements, has significantly influenced treatment outcomes in vital pulp therapy (VPT) procedures [19, 20]. These materials demonstrate superior biocompatibility and the ability to stimulate reparative dentinogenesis compared to traditional materials [21, 22]. However, questions remain regarding optimal clinical protocols, case selection criteria, and long‐term outcomes. Treatment approaches must be adapted for different patient populations and tooth types.

Pulp therapy in primary teeth presents unique challenges due to their limited lifespan and the presence of a developing permanent successor [2]. The management of immature permanent teeth requires special consideration due to their thin dentinal walls and open apices, necessitating approaches that can promote continued root development [23].

### 1.1 Scope and Purpose

This research paper formulates consensus‐based recommendations for managing pulp therapies in primary and permanent teeth. Specifically, this paper:

1. Synthesizes the current evidence regarding the biological basis for VPT.
2. Provides evidence‐based guidelines for diagnosis and appropriate selection for various pulp therapy procedures in primary and permanent teeth.
3. Identifies research gaps and future directions for advancing evidence‐based care.

### 1.2 Target Users

The target users are dental professionals including general practitioners, pediatric dentists, and endodontists.

### 1.3 Target Population

The target population includes children and adolescents requiring pulp treatment due to caries or trauma in primary and permanent teeth.

### 1.4 Exceptions

Clinicians may need to modify these recommendations due to various factors. These include the patient's cooperation level, complex medical conditions, difficulty achieving local anesthesia, limited mouth opening, severe gag reflex, facial swelling, undiagnosed oral pain, complications from previous pulp therapy, or concurrent periodontal issues. Individual considerations such as parent and patient preferences, patient's age, and treatment costs may necessitate alternate treatment approaches including extraction of the tooth.

## 2 Materials and Methods

**FIGURE 1:**
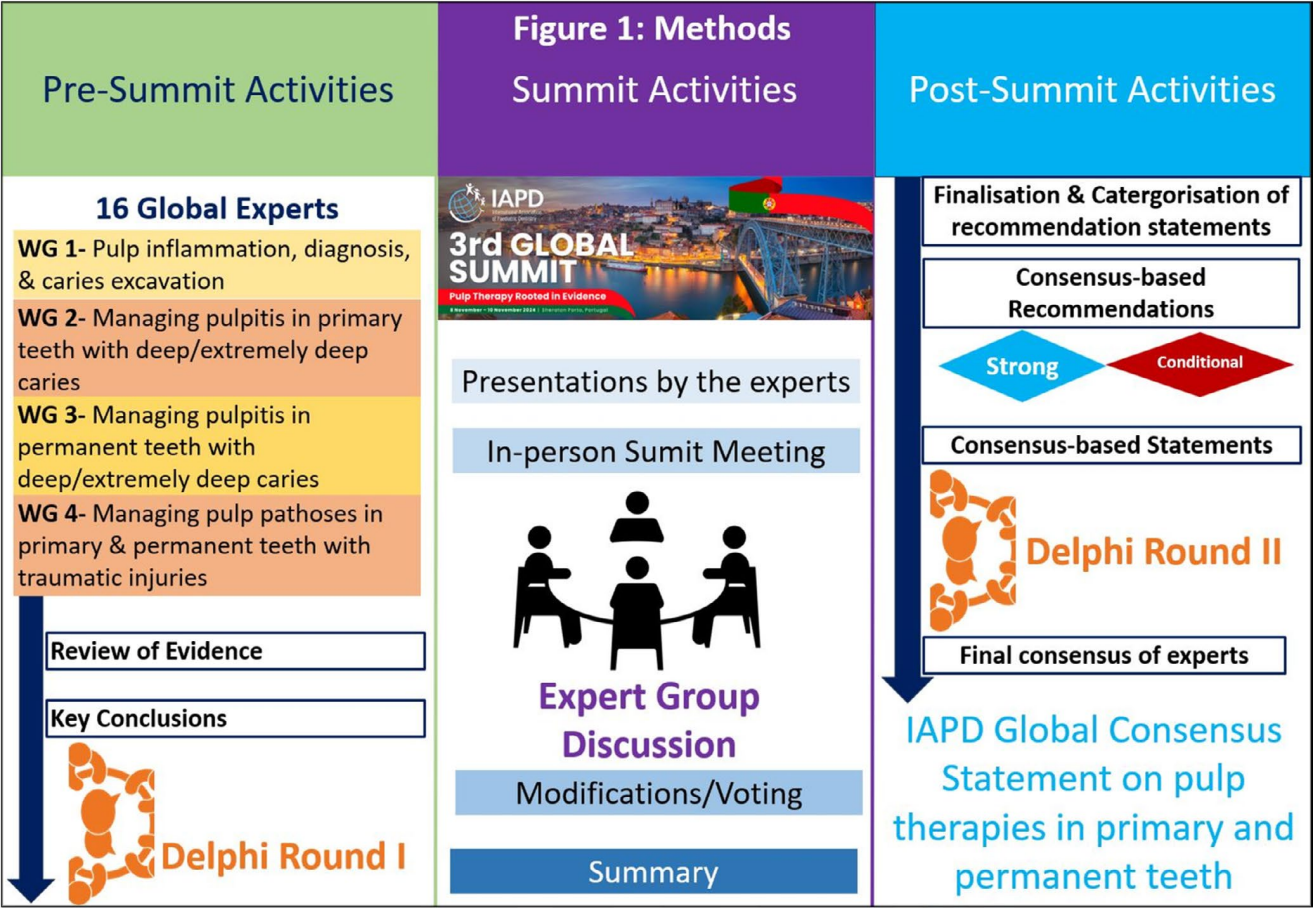
Methods.

These consensus‐based recommendations were derived from 16 international specialists in pediatric dentistry (10 experts) and endodontics (6 experts). Some of these experts also had internationally recognized expertise in cariology. The experts convened at the 3rd IAPD Summit in Porto, Portugal, in November 2024. They followed a structured approach comprising three phases: pre‐summit preparation, summit meeting, and post‐summit activities (Figure 1).

### 2.1 Pre‐Summit Activities

First, the working groups (WG) comprised 16 experts, was established and assigned to focus on four key areas in pulp therapy (Table 1). Each expert panelist systematically searched the literature of their area of expertise. They submitted draft recommendations 2 months before the summit. From these drafts, 38 recommendation statements were created that underwent a Delphi process using a 7‐point Likert Scale.

**TABLE 1:** Workgroups (WGs).

**Expert Panel Chair:** Vineet Dhar
**WG 1:** Pulp inflammation, diagnosis, and caries excavation
Members: Ikhlas El‐Karim (Chair), James A. Coll, Ashraf Fouad, Nicola Innes, Jonas A. Rodrigues
**WG 2:** Managing pulpitis in primary teeth with deep/extremely deep caries
Members: James A. Coll (Chair), Yasmi Crystal, Zafer Çehreli, Vineet Dhar, Mani Ekambaram, Brian Hodgson
**WG 3:** Managing pulpitis in permanent teeth with deep/extremely deep caries
Members: Ashraf Fouad (Chair), Saeed Asgary, Lars Bjorndal, Nessrin Taha, Tugba Turk
**WG 4:** Managing pulp pathoses in primary and permanent teeth with traumatic injuries
Members: Anne O'Connell (Chair), Nitesh Tewari

The Delphi process is a structured method that uses multiple rounds of anonymous surveys with a panel of experts, where participants refine their opinions based on feedback from previous rounds until consensus emerges. It is designed to harness collective expert judgment while avoiding the biases of face‐to‐face group dynamics [24].

### 2.2 Summit Activities

During the meeting, the WGs made minor editorial edits to recommendations with over 70% agreement and revised the recommendations that received under 70% agreement in the initial Delphi round. The WGs created: consensus‐based recommendations and consensus‐based statements, both developed with Delphi agreement. The recommendations were categorized based on the strength of supporting evidence. Strong recommendations were based on randomized controlled trials (RCTs), systematic reviews, or clinical practice guidelines. Conditional recommendations were based on lower‐quality studies such as observational studies, while consensus‐based statements reflected expert opinion.

Consensus‐based recommendations drawn from clinical practice guidelines were evaluated for strength according to their original source and were not automatically elevated to the “strong” category.

### 2.3 Post‐Summit Activities

After a second Delphi survey, all recommendations received over 70% consensus from the 16‐member workgroup. This resulted in 38 recommendations that were reviewed and approved by the WGs in the final survey. The glossary of terms is presented in Table 2.

**TABLE 2:** Glossary of terms.

**Deep caries:** Carious lesions extending into two‐thirds of dentin thickness (inner third) in primary teeth and three‐fourths of dentin thickness (inner quarter) in permanent teeth, with a definitive radiographic dentin barrier. These lesions can be categorized as ICDAS 5 lesions, are close to pulp, and at risk for pulp exposure with non‐selective removal of carious tissue.
**Extremely deep caries:** Carious lesions which penetrates the entire radiographic thickness of dentin with no discernable radiographic dentin barrier, indicating pulp exposure would be unavoidable.
**ICDAS 5:** A distinct clinical cavitation in opaque or discolored enamel with visible dentin and radiolucency reaching the inner one‐third of dentin.
**Reactionary dentin:** Dentin produced by stimulated odontoblasts responding to mild inflammation caused by bacterial byproducts diffusing through tubules from slow‐progressing caries.
**Reparative dentin:** Dentin produced by odontoblast‐like cells differentiated from dental pulp stem cells responding to controlled inflammation following severe pulp injury and odontoblast cell death.
**Reversible pulpitis:** A clinical diagnosis suggestive of early stages of pulpal inflammation characterized by brief, non‐spontaneous discomfort triggered by cold or sweet stimuli that resolves within seconds of stimulus removal, indicating that the pulp is capable of healing completely. (AAE's Reversible Pulpitis/Wolter's initial to mild pulpitis criteria.)
**Irreversible pulpitis:** A clinical diagnosis indicating that the vital inflamed pulp is incapable of healing, characterized by spontaneous pain, sharp and lingering pain triggered by thermal stimuli, and referred pain. (AAE's Irreversible Pulpitis/Wolter's moderate to severe pulpitis criteria.)
**Selective caries removal to soft dentin**: Selective removal of carious tissue in deep lesions, which includes leaving soft carious tissue over the pulp to avoid exposure and stress to pulp, while preparing up to hard dentin peripherally, with the aim of achieving a well‐sealed restoration.
**Non‐selective (complete) caries removal:** Removal of all carious tissue leaving hard dentin behind.
**Stepwise caries removal:** Removal of carious tissue in two stages. In first visit, soft carious tissue is left over the pulp, while peripheral dentin is prepared to hard dentin to allow a complete and durable seal of the lesion with a provisional restoration. In the second visit, restoration is reentered with removal of remaining carious tissue until “leathery” dentin remains over the pulp, followed by a definitive restoration.
**Hall technique:** Treatment for a decayed tooth where no decay is removed, and a preformed metal crown is cemented with glass ionomer cement to minimize microleakage and stop the progression of dental caries.
**Indirect pulp treatment (IPT):** According to AAPD's Clinical Practice Guidelines, IPT involves selective removal of carious tissue to prevent pulp exposure, followed by the placement of a liner and a well‐sealed restoration.
**Direct pulp capping (DPC):** Placement of a biocompatible material over a clinically visible pulp exposure caused by trauma or at the time of caries removal, followed by a well‐sealed restoration.
**Partial pulpotomy (PP):** Incremental removal of the affected portion of the vital coronal pulp, followed by the placement of a biocompatible material over the remaining pulp, and covered with a well‐sealed restoration.
**Full pulpotomy (FP):** Complete removal of the coronal pulp, followed by the placement of a biocompatible material over the radicular pulp, and covered with a well‐sealed restoration.
**Non‐instrumental endodontic treatment (NIET) or lesion sterilization and tissue repair (LSTR):** It is a short‐term treatment for necrotic primary teeth that usually requires no instrumentation of the root canals or obturation of the canals but instead includes the placement of an antibiotic mixture in the pulp chamber to disinfect the root canals.
**Concomitant luxation injury:** Injury to the supporting structures (periodontium) that occur in addition to an injury to hard dental tissue. These occur in a single tooth or may affect multiple teeth. Some of the authors have identified this condition as combination dental injuries.
**External inflammatory root resorption:** An inflammatory process that occurs when a tooth has an infected root canal system and there has been damage to the external surface of the tooth root (i.e., cementum) or to the periodontal ligament during a traumatic dental injury (such as luxation or avulsion), or when there is a pathway of communication between the root canal system and the peri‐radicular tissues (such as, via the apical foramen or a lateral canal) and bacteria and/or their endotoxins are able to escape into the peri‐radicular tissues to induce inflammation resulting in root resorption.
**External replacement resorption:** The process that occurs when the periodontal ligament and/or cementum root surface have been damaged, particularly after luxation or avulsion injuries and where the cementum and dentin are resorbed and replaced by bone over time. It follows ankylosis, and it is a progressive process that will eventually involve the entire root.
**Pulp canal obliteration:** Radiographic evidence of increased dentin production primarily in response to trauma. The end result of this process is a calcified canal and is not necessarily indicative of a diseased pulp.

### 2.4 Understanding the Recommendations/Statements

The recommendations are intended to assist clinicians in making informed decisions regarding pulp therapies. They do not replace clinical judgment.

Strong recommendations for specific interventions indicate that the WGs have determined that the benefits substantially outweigh potential risks. In these cases, clinicians should consider implementing the recommended intervention as a standard approach.

Conditional recommendations reflect that the WG felt less certain in the benefit–risk assessment. These recommendations may change with new evidence. A conditional recommendation suggests that while an intervention may be appropriate for some patients, clinicians should carefully evaluate individual circumstances and consider alternative approaches.

Consensus‐based statements represent expert‐derived best practices in areas where high‐quality research evidence is currently limited or unavailable.

## 3 Results

**FIGURE 2:**
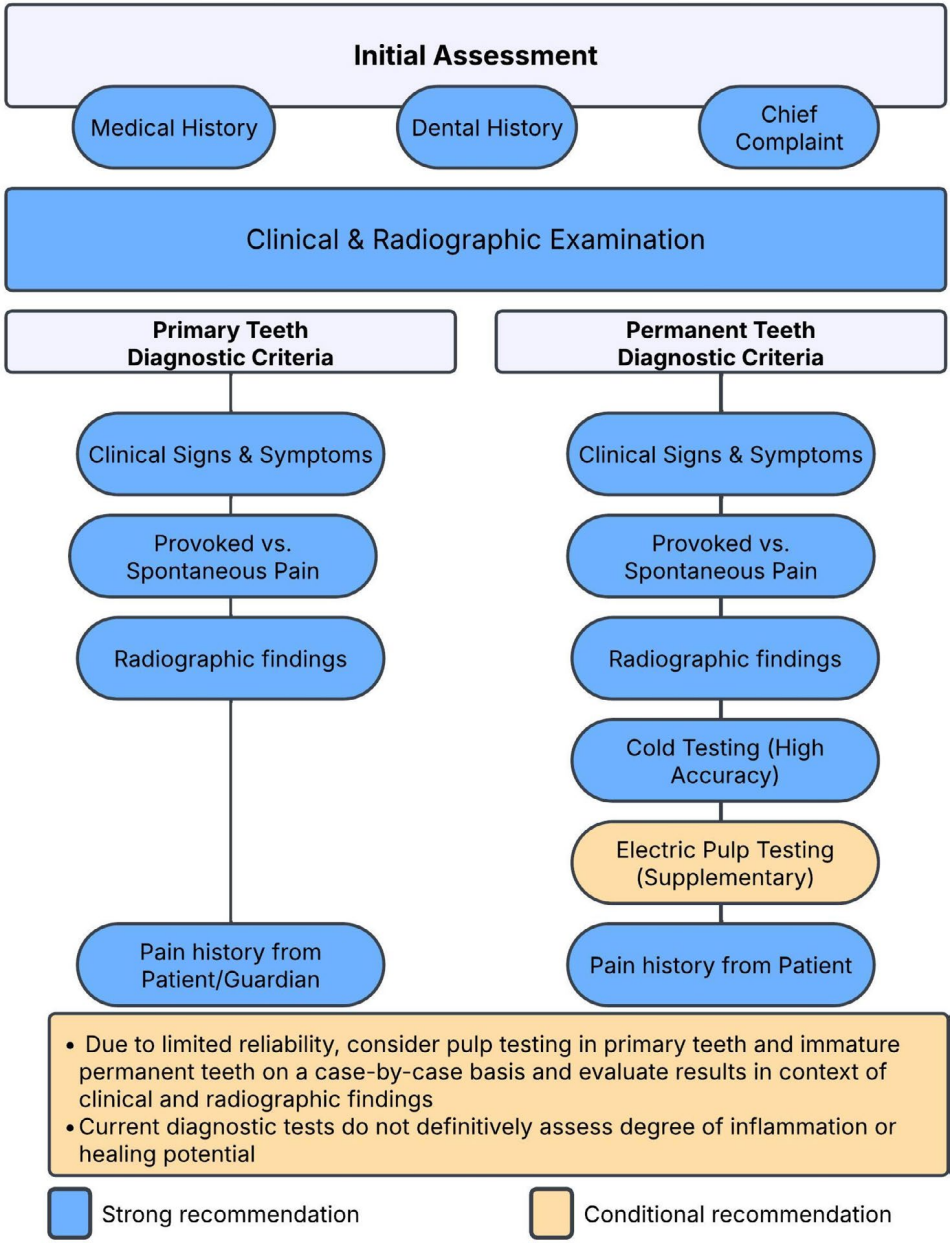
Diagnostic approach to pulpal status assessment.

**FIGURE 3:**
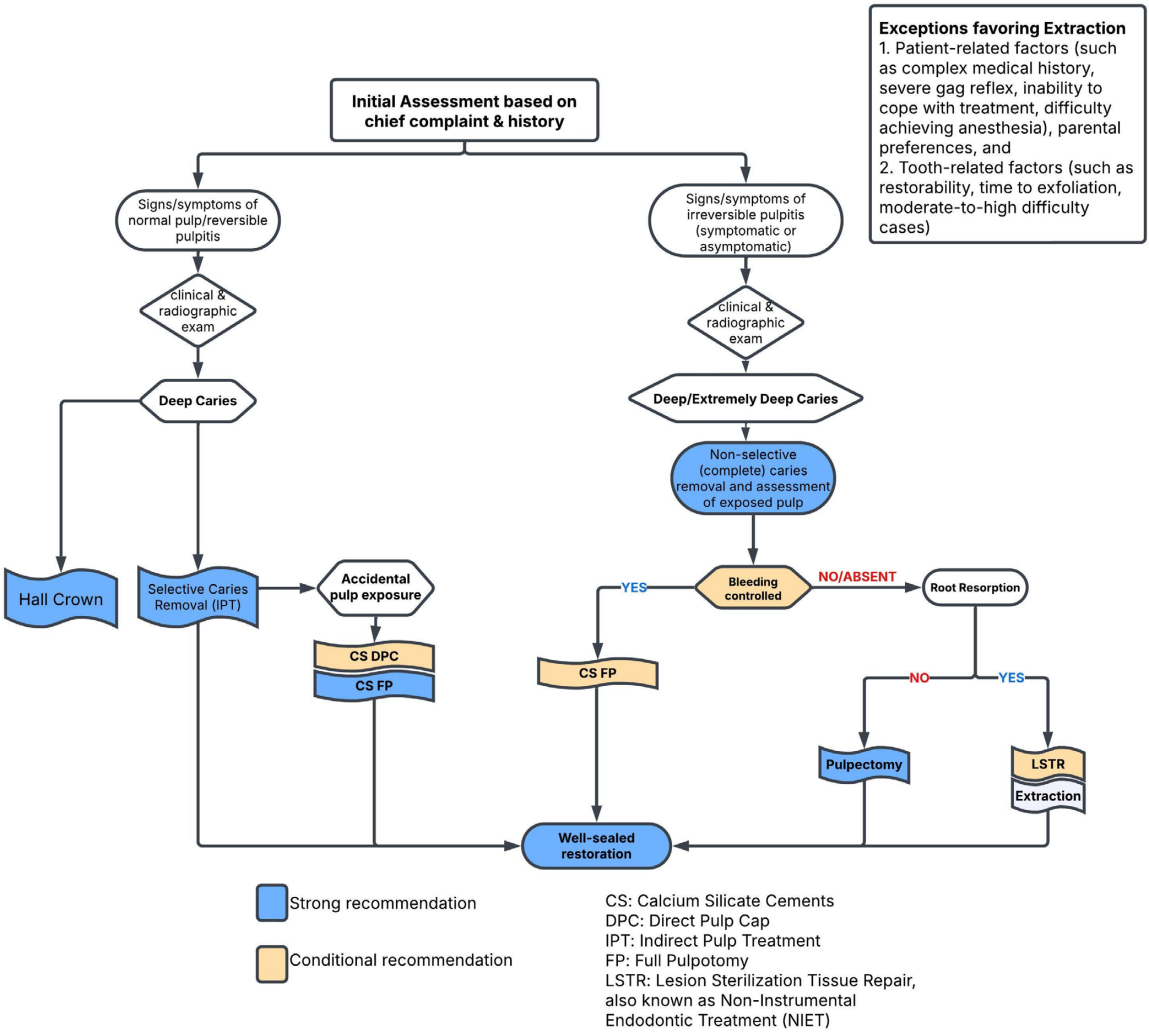
Management of pulpitis in carious primary teeth.

**FIGURE 4:**
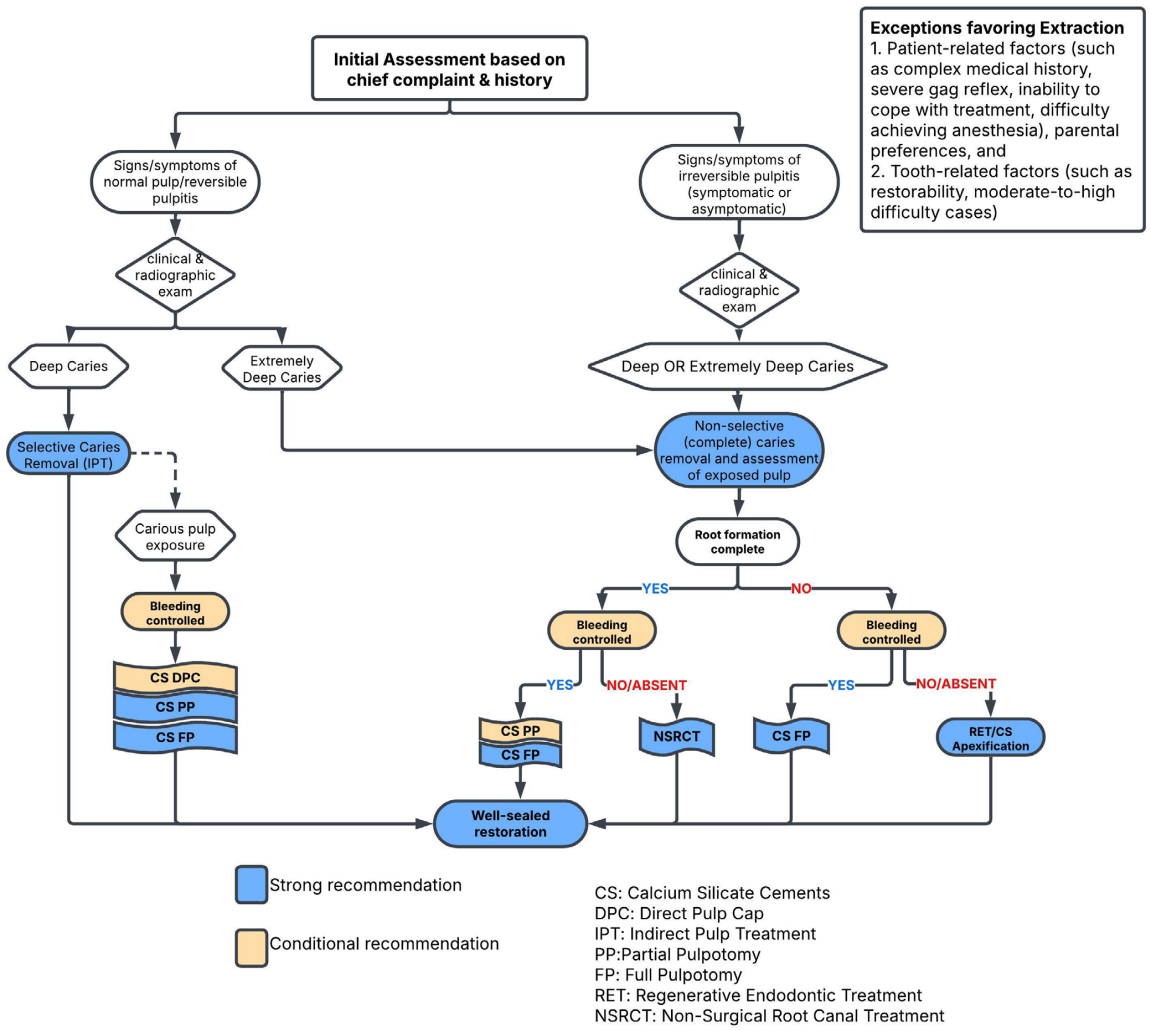
Management of pulpitis in carious permanent teeth.

**FIGURE 5:**
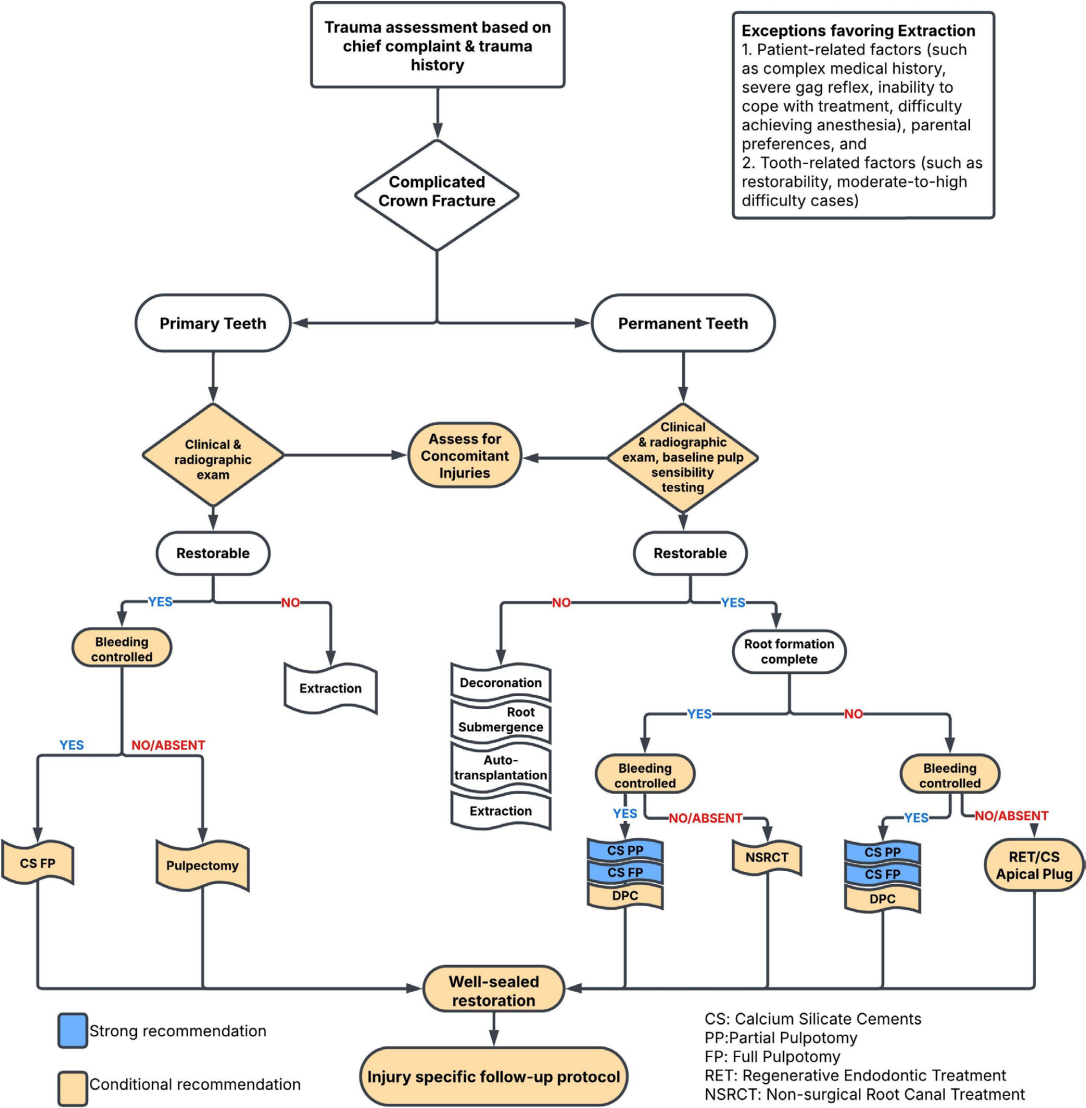
Management of complicated crown fracture.

The 38 recommendations regarding pulp therapies in primary and permanent teeth, along with their strength and percent agreement, are presented (Tables 3, 4, 5, 6). The WGs have also developed evidence‐based decision trees on pulp diagnosis and treatment to support clinicians in their chairside decision‐making (see Figures 2, 3, 4, 5).

**TABLE 3:** Pulp inflammation, diagnosis, and caries excavation.

	Statement	Type of statement	Strength of evidence (percent agreement)
**Pulp inflammation & diagnosis**			
1.	In primary and permanent teeth with deep caries diagnosed with reversible pulpitis, clinicians should attempt conservative treatment, as the inflamed dental pulp, if not exposed, can heal when provided with a favorable environment. In these cases, the inflammatory process stimulates odontoblasts to produce reactionary dentin.	Consensus‐based Recommendation	Conditional (> 70%)
2.	In primary and permanent teeth with extremely deep caries and iatrogenic pulp exposure, severe injury causes odontoblast cell death and the inflammatory response triggers differentiation of the dental pulp stem cells to produce reparative dentin, if an environment favorable for healing can be created.	Consensus‐based Recommendation	Conditional (> 70%)
3.	Pulpal diagnosis requires a comprehensive assessment and documentation of the patient's medical and, dental history, chief complaint, along with the results of diagnostic tests, and clinical and radiographic examinations. Key signs and symptoms to be considered include the onset, course, duration, intensity, aggravating and relieving factors, nocturnal pain, as well as evoked and persistent pain. For permanent teeth, the painful response and duration to cold stimuli should be assessed. For primary teeth, clinicians should primarily rely on clinical signs and symptoms of provoked versus spontaneous pain, along with radiographic findings.	Consensus‐based Recommendation	Conditional (> 70%)
4.	In mature permanent teeth, cold testing exhibits high sensitivity, specificity, and accuracy, even in crowned teeth. It can be complemented by electric pulp testing when the results are inconclusive. Given its limited reliability, diagnostic pulp testing should be carried out on a case‐by‐case basis in primary teeth and immature permanent teeth, using clinical judgment to interpret results.	Consensus‐based Recommendation	Conditional (> 70%)
5.	Current diagnostic tests cannot definitively assess the degree of inflammation or the healing potential of the dental pulp in both primary and permanent teeth. Although there are no commercially available devices for clinical use, the assessment of the pulp's microcirculation and vitality may be determined by measuring oxygen saturation. Currently, the biomarkers of inflammation have limited utility in clinical practice due to lack of established thresholds.	Consensus‐based Recommendation	Conditional (> 70%)
6.	The duration of pulpal bleeding should be considered alongside other diagnostic indicators of pulpal health rather than in isolation. Although prolonged bleeding has not been definitively established as a predictor of outcomes of VPT, achieving hemostasis may be viewed to be of practical and pragmatic importance for the success of VPT.	Consensus‐based Recommendation	Conditional (> 70%)
7.	In primary teeth with deep caries, clinicians may consider placing a temporary glass ionomer restoration without any caries tissue removal or after selective caries removal to soft dentin (interim therapeutic restoration) for a duration of 1–3 months as a diagnostic tool for pulp vitality before proceeding with definitive treatment. The interim restoration must provide an adequate seal to prevent bacterial ingress while monitoring pulpal response.	Consensus‐based Recommendation	Conditional (> 70%)
8.	For the management of deep and extremely deep carious lesions, particularly in cases of pulp exposure, it is suggested to utilize magnification for direct visualization of the pulp, including pulpal bleeding, homogeneity of exposure, and to identify any areas of necrosis and degeneration.	Consensus‐based Statement	Strength—not determined (> 70%)
**Caries excavation**			
9.	After confirming pulp vitality and considering the stage of tooth development, as well as the ability to place a well‐sealed restoration, clinicians should adopt a conservative approach to managing deep caries in both primary and permanent teeth. It is recommended to use selective caries removal or stepwise removal over non‐selective (complete) caries removal, except in cases of extremely deep caries. Selective caries removal offers an additional advantage as it is completed in a single visit.	Consensus‐based Recommendation	Strong (> 70%)
10.	For vital primary teeth with multi‐surface or large single‐surface deep carious lesions, either no carious tissue removal with Hall Technique or selective caries removal to soft dentin followed by a preformed crown is recommended over non‐selective (complete) caries tissue removal.	Consensus‐based Recommendation	Strong (> 70%)
11.	Considering the risk of pulp exposures, treatment of deep and extremely deep carious lesions should be performed using a dental dam to isolate the lesion and protect against bacterial contamination. Dental dam isolation is not needed when using Hall technique for management of deep caries in primary teeth.	Consensus‐based Statement	Strength—not determined (> 70%)

**TABLE 4:** Managing pulpitis and pulp necrosis in primary teeth.

	Statement	Type of statement	Strength of evidence (percent agreement)
12.	For vital primary teeth with deep caries, in the event of a pulp exposure, clinicians should utilize calcium silicate cement (CSC) as VPT medicament. These biomaterials promote pulp healing, stimulate the formation of reparative dentin, provide an effective seal, and thereby enhance the long‐term success of the treatment.	Consensus‐based Recommendation	Strong (> 70%)
13.	When evaluating for VPT, selective caries removal (IPT) should be the preferred approach for primary teeth with deep caries and healthy pulp or reversible pulpitis due to its high success rate.	Consensus‐based Recommendation	Strong (> 70%)
14.	DPC is a viable treatment option for managing pulp exposures measuring less than 1 mm in vital primary teeth with deep caries diagnosed with healthy pulp or reversible pulpitis. However, DPC has a lower success rate compared to pulpotomy.	Consensus‐based Recommendation	Conditional (> 70%)
15.	Clinicians should consider performing FP using CSCs in the event of pulp exposure in primary teeth with deep caries and diagnosed with healthy pulp or reversible pulpitis.	Consensus‐based Recommendation	Strong (> 70%)
16.	Primary teeth that exhibit signs of irreversible pulpitis, based on history of spontaneous pain, can be managed successfully with a CSC pulpotomy for 12 months or possibly longer, provided the pulp upon access appears to be vital and there are no signs of necrosis or furcal radiolucency. Achieving hemostasis is considered important for the success of this CSC pulpotomy.	Consensus‐based Recommendation	Conditional (> 70%)
17.	Pulpectomy can be considered as a non‐vital pulp treatment (NVPT) option for primary teeth without preoperative root resorption diagnosed with irreversible pulpitis or necrotic pulp with or without furcal radiolucency. This procedure can be effectively performed using either rotary or manual instrumentation, along with irrigation solutions such as sodium hypochlorite (1%–5%), saline, or chlorhexidine (2%).	Consensus‐based Recommendation	Strong (> 70%)
18.	When obturating root canal(s) of a primary tooth undergoing pulpectomy, select either Zinc Oxide/Iodoform/Calcium Hydroxide or Zinc Oxide Eugenol (ZOE), over Calcium Hydroxide/Iodoform as filling materials based on 18‐month success rates. To avoid overfilling, utilize pluggers for ZOE and lentulo spirals for calcium hydroxide/Iodoform‐based materials.	Consensus‐based Recommendation	Conditional (> 70%)
19.	Non‐Instrumental Endodontic Treatment (NIET), also referred to as Lesion Sterilization and Tissue Repair (LSTR), may be an alternative to pulpectomy for primary teeth with preoperative root resorption diagnosed with irreversible pulpitis or necrotic pulp knowing it has only shown short‐term success and has the potential for antibiotic resistance associated with the procedure.	Consensus‐based Recommendation	Conditional (> 70%)
20.	Following a pulpotomy, NIET (LSTR), or pulpectomy, clinicians should restore the treated tooth with a preformed crown to protect against reinfection and enhance the tooth's long‐term survival.	Consensus‐based Recommendation	Conditional (> 70%)

**TABLE 5:** Managing pulpitis and pulp necrosis in permanent teeth.

	Statement	Type of statement	Strength of evidence (percent agreement)
21.	For VPT in permanent teeth with deep caries, clinicians should utilize CSCs. These biomaterials promote pulp healing and stimulate the formation of reparative dentin, thereby enhancing the long‐term success of the treatment. Glass ionomer cement may be used in absence of pulp exposure. Placement of a well‐sealed coronal restoration enhances the long‐term success of the treatment.	Consensus‐based Recommendation	Strong (> 70%)
22.	Selective caries removal (as known as IPT), should be considered as a viable option for managing deep caries, but not extremely deep caries, in permanent teeth diagnosed with healthy or reversible pulpitis.	Consensus‐based Recommendation	Strong (> 70%)
23.	DPC is a viable treatment option for cases of pulp exposure while removing deep caries in permanent teeth, particularly when the pulp is diagnosed as healthy or reversibly inflamed. Notably, the long‐term success rate of DPC in permanent teeth is higher when calcium silicate cements are used as a medicament.	Consensus‐based Recommendation	Conditional (> 70%)
24.	For the management of pulp exposure in permanent teeth with extremely deep caries and various degrees of pulpitis, clinicians can perform either a PP or FP using CSCs, as this approach has demonstrated a high success rate. If hemostasis cannot be achieved when attempting a partial pulpotomy, further tissue removal may be necessary by deepening the partial pulpotomy or progressing to a full pulpotomy.	Consensus‐based Recommendation	Conditional (> 70%)
25.	Due to its minimally invasive nature and potential for pulp preservation, full pulpotomy using CSCs can be an alternative treatment to non‐surgical root canal treatment for mature permanent teeth with carious pulp exposure and clinical signs and symptoms of irreversible pulpitis, such as spontaneous or lingering pain, provided that there are no signs and symptoms of pulp necrosis. Achieving hemostasis may be viewed to be important for the success of VPT. The success rate of this full pulpotomy may be comparable to that of root canal treatment for vital permanent teeth.	Consensus‐based Recommendation	Strong (> 70%)
26.	For immature permanent teeth with extremely deep caries diagnosed with irreversible pulpitis and there are no signs and symptoms of pulp necrosis, FP using CSCs is the recommended VPT due to its effectiveness in supporting root development and maintaining pulp vitality.	Consensus‐based Recommendation	Strong (> 70%)
27.	Both apexification and Regenerative Endodontic Treatments (RETs) are viable options for managing immature, necrotic permanent teeth. The use of CSCs, which are supported by evidence, is preferred over calcium hydroxide (CH) apexification.	Consensus‐based Recommendation	Strong (> 70%)

**TABLE 6:** Managing pulp pathoses in primary & permanent teeth with traumatic injuries.

	Statement	Type of statement	Strength of evidence (percent agreement)
28.	Traumatic dental injuries affect the dental pulp either directly or indirectly due to infection, inflammation, and damage to the periodontium. Evidence supports that dental pulp tissue can heal after dental trauma; therefore clinicians should allow time for healing especially in cases of mild injury, immature teeth, and younger patients.	Consensus‐based Recommendation	Conditional (> 70%)
29.	Traumatic dental injuries must be documented in a standardized manner and validated objective criteria must be used for interpreting the radiographic features for clearer prognostic evaluation.	Consensus‐based Recommendation	Conditional (> 70%)
30.	Clinicians should evaluate for concomitant luxation injuries as these can cause disruption of the vascular supply to the pulp negatively impacting healing in immature permanent teeth with crown fractures.	Consensus‐based Recommendation	Conditional (> 70%)
31.	Clinicians should use a combination of clinical and radiographic signs and symptoms such as pain, infection, discoloration, mobility, and pathological changes in the tooth or bone to assess the need for endodontic intervention in teeth with traumatic injuries. Discoloration in both dentitions may be transient and thereby not a reliable indicator of necrosis. In addition, responses to pulp sensibility tests (thermal, electric, and occasionally, cavity tests) may assist with diagnosis of necrotic pulp in permanent teeth. For primary teeth, pulp sensibility tests are not indicated. A positive response to sensibility tests shortly after trauma is a good predictor of vitality but lack of response is not always associated with subsequent necrosis, especially luxation injuries.	Consensus‐based Recommendation	Conditional (> 70%)
32.	PP or FP has a high success rate in complicated crown fractures. Direct pulp capping has a lower success rate in permanent teeth with complicated crown fractures but may be suitable in select cases or as a temporary treatment in uncooperative children until pulpotomy can be performed. Clinicians should use non‐staining CSCs to prevent discoloration.	Consensus‐based Recommendation	Strong (Pulpotomy) Conditional (DPC) (> 70%)
33.	Appropriate immediate management can limit the adverse complications of most traumatic injuries; however, avulsion and severe luxation (intrusive, extrusive, and lateral) injuries are associated with a higher incidence of complications.	Consensus‐based Recommendation	Conditional (> 70%)
34.	Appropriate follow up of traumatic injuries is important due to the risk of late complications such as discoloration, pulp necrosis, arrest of root formation, apical periodontitis, external inflammatory root resorption, external replacement (ankylosis‐related) resorption, external invasive resorption, and internal resorption. The incidence and management of these complications differ between primary and permanent teeth.	Consensus‐based Recommendation	Conditional (> 70%)
35.	Traumatized immature permanent teeth with pulp necrosis can be managed either by RET or by apical plug using CSC barrier. Both methods have demonstrated comparable clinical, radiographic, and overall success and survival rates. However, RET may be preferred for necrotic teeth in very early stages of root development and for teeth with developmental dental anomalies.	Consensus‐based Recommendation	Conditional (> 70%)
36.	External inflammatory root resorption can be arrested by careful removal of causative factors by endodontic debridement, irrigation, and use of intracanal medicament (Calcium Hydroxide, Triple Antibiotic Paste, Double Antibiotic Paste). External replacement resorption is progressive in nature and cannot be stopped or reversed. In permanent teeth, decoronation or root submergence or autotransplantation and extraction are acceptable treatments in suitable patients.	Consensus‐based Recommendation	Conditional (> 70%)
37.	Pulp canal obliteration in primary and permanent teeth is a healing response and yellow discoloration is due to the internal layers of dentin formed in response to injury. Clinicians should monitor these teeth without any intervention unless pulp necrosis and apical periodontitis develop.	Consensus‐based Recommendation	Conditional (> 70%)
38.	In traumatized permanent incisors, the use of non‐surgical endodontic protocols for teeth with symptomatic or asymptomatic apical periodontitis or acute abscess with varying grades of periapical radiolucency is indicated in cases that fail to respond to RET.	Consensus‐based Recommendation	Conditional (> 70%)

A summary of the supporting evidence along with justification for the assigned strength of evidence is outlined in the Appendix S1.

## 4 Discussion

Conducting high quality research on pulp therapies in children presents several methodological challenges. These include navigating Institutional Review Board approval processes for studies involving minors, collecting long‐term follow‐up data, and working with small sample sizes due to the infrequency of severe pediatric dental trauma cases. This highlights the need for multi‐institutional collaboration, innovative study designs that make optimal use of limited cases, and sustained funding to support the long follow‐up times necessary to confirm outcomes of pediatric dental trauma.

Despite these obstacles, ongoing efforts to develop creative methodologies and strengthen research networks remain important for advancing evidence‐based care. The review of current evidence has identified research gaps and future directions:

Evidence suggests that current categories of reversible and irreversible pulpitis are inadequate to reflect the capacity of the pulp to heal and should be modified [7, 10]. A critical research priority remains the development of reliable, objective diagnostic tools for assessing pulpal status. While current diagnostic methods rely heavily on clinical symptoms and sensibility testing, there is a pressing need for chairside diagnostic tests and biomarkers that can accurately reflect the inflammatory status of the pulp [14, 16]. Future research should focus on establishing clearer correlations between clinical symptoms and histological status of the pulp, as current evidence shows significant discrepancies between these parameters [25]. Research is also needed to better understand the optimal endpoints for caries removal in deep lesions, particularly regarding the biological markers that indicate successful conservative treatment. Long‐term studies comparing outcomes between selective and complete caries removal approaches are essential, especially given the paradigm shift toward more conservative techniques [16, 26].

Research in primary tooth pulp therapy faces unique challenges requiring specific attention. There is a pressing need for age‐appropriate diagnostic methods, as current pulp testing approaches in caries and trauma show limited reliability in young children largely due to difficulty in performing and interpreting children's responses [2, 27]. Future studies should focus on developing diagnostic criteria specifically tailored to primary teeth, considering their unique anatomical and physiological characteristics. Long‐term outcome studies comparing different VPT techniques are crucial, particularly regarding the efficacy of newer bioactive materials in primary teeth. Research should also investigate the impact of child‐specific factors such as age, behavior, and compliance on treatment outcomes. The development of biocompatible materials specifically designed for primary teeth represents another important research direction, as current materials are often adaptations of those developed for permanent teeth.

Research priorities for VPT in permanent teeth should focus on generating high‐quality evidence to improve clinical outcomes. Extended longitudinal studies (5–10 years) comparing VPT outcomes with conventional root canal treatment are critically needed in children and adolescents, where maintaining tooth vitality for decades is essential [28]. While current evidence supports using vital pulp therapies in cases previously considered irreversible, robust long‐term comparative data would help establish definitive clinical protocols [18, 29, 30]. Standardized, evidence‐based protocols addressing specific indications for each VPT technique, optimal irrigation solutions, hemostasis protocols, and restoration techniques would enhance clinical predictability [31, 32]. More studies directly comparing different VPT approaches (IPT, DPC, PP, and FP) with standardized methodologies, outcomes, and outcome measures would help establish a hierarchy of treatment options based on tooth type, patient age, extent and location of caries, and preoperative symptoms [16, 33]. Given these limitations, permanent teeth that have undergone VPT, which includes IPT, should have long‐term monitoring during recall visits and routine radiographic examinations to facilitate timely intervention or referral when indicated. Research should investigate materials and medications that are specifically anti‐inflammatory and therapeutic to the inflamed pulp, beyond current CSCs [34]. This includes exploration of pharmacological agents that can effectively modulate pulpal inflammation, bioactive materials that promote pulp healing and dentinogenesis, and novel delivery systems that optimize therapeutic effects at the pulp‐dentin interface [35, 36].

The identification and validation of reliable biomarkers for pulpal inflammation would significantly improve diagnostic accuracy and treatment planning [37]. Investigation into pulp regeneration strategies incorporating tissue engineering principles could potentially transform management of deeply carious permanent teeth [1]. This includes research on stem cell applications, growth factors that promote pulpal healing, and biomimetic scaffolds that support tissue regeneration [23, 38, 39]. By addressing these research priorities, the profession can continue to advance toward more predictable, biologically based approaches to managing pulpal disease in permanent teeth, potentially reducing the need for more invasive procedures while maintaining pulp vitality for improved long‐term outcomes [40].

Future research in dental trauma should prioritize the development of improved methods for assessing pulp status following injury in both primary and permanent teeth. This is particularly important in the immediate post‐trauma period when current diagnostic tools may be unreliable. Studies are needed to identify early indicators of pulp necrosis and better understand healing patterns in different types of traumatic injuries. Age‐related differences in healing responses require further investigation, especially in determining the optimal timing of intervention after trauma. Research into regenerative approaches for traumatized immature permanent teeth is particularly important, as these cases present unique challenges and opportunities for promoting continued root development [23]. Several research priorities span across all areas of pulp therapy. The development of standardized outcome measures and uniform reporting criteria for clinical studies is essential for advancing the field. The need for a well‐sealed restoration following vital pulp therapy is universally recognized; however, there is a critical need to deepen our understanding of the molecular and cellular mechanisms underlying pulp‐dentin complex responses to injury and treatment. Integrating new technologies, such as advanced imaging techniques and artificial intelligence applications into diagnostic practices offers promising directions for future research. The benefit of cone beam computed tomography (CBCT) should be assessed and referral to a specialist made when appropriate. Investigation into cost‐effectiveness and long‐term outcomes of conservative versus invasive approaches is needed to inform clinical decision‐making. Future studies should also focus on developing minimally invasive treatment approaches and novel delivery systems for therapeutic agents, while further advancing our understanding of regenerative techniques and biomaterial development [4, 5]. These research priorities highlight the need for a multi‐faceted approach to advancing the field of pulp therapy, combining basic science research with clinical investigations to develop more predictable and less invasive treatment approaches in both primary and permanent teeth. Success will require collaboration across disciplines and continued investment in both laboratory and clinical research infrastructure.

In summary, clinicians often encounter uncertainty in diagnosing the condition of pulp, as definitive histological confirmation of pulpitis is not feasible. While clinical symptoms, radiographic signs, and pulp sensibility tests offer guidance, they do not consistently reflect the healing potential of the inflamed pulp. Pulpal inflammation should be considered a continuum rather than a binary condition. In cases where teeth exhibit clinical signs and symptoms of pulpitis, treatment should be initiated using a tiered approach, with conservative methods as the first‐line treatment. Advancing our understanding of pulp biology, refining diagnostic tools, and developing more conservative and regenerative therapies are essential to improving long‐term outcomes and preserving natural dentition. The IAPD Porto Forum has outlined key research priorities to address the current challenges and unmet needs in managing pulp inflammation and trauma in primary and permanent teeth. By addressing these identified research priorities, the dental community can work toward a future where the inflamed or traumatized pulp is managed with greater precision, reliability, and biological compatibility. This will ultimately lead to better quality of life for patients of all ages.

## Author Contributions

Dr. Vineet Dhar led the expert panel. Dr. Ikhlas El‐Karim, Dr. James A. Coll, Dr. Ashraf F. Fouad, and Dr. Anne C. O'Connell led workgroups on specific domains. All authors contributed equally to concept, writing, and revisions.

## Funding

The authors have nothing to report.

## Conflicts of Interest

The authors declare no conflicts of interest.

## Supporting information


**Data S1:** ipd70068‐sup‐0001‐AppendixS1.pdf.

## Data Availability

The data that support the findings of this study are available on request from the corresponding author. The data are not publicly available due to privacy or ethical restrictions.
