# Supplementary material for: Consensus‐Based Recommendations on Pulp Therapies in Primary and Permanent Teeth: IAPD Porto Forum
Source: Int J Paediatr Dent. 2026 Feb 2;36(2):371–86. doi: 10.1111/ipd.70068 (PMC12916461; doi:10.1111/ipd.70068)
Supplement: Supplementary file 1 — Data S1: ipd70068‐sup‐0001‐AppendixS1.pdf. [file IPD-36-371-s001.pdf]

Consensus-based Recommendations on Pulp Therapies in Primary and Permanent Teeth: IAPD Porto Forum

Appendix S1: SUMMARY OF EVIDENCE

Working Group 1: Pulp Inflammation, Diagnosis, and Caries Excavation

WG 1.1 Pulp Inflammation & Diagnosis (Table 3)

1. Pulpal Response to Deep Caries in Primary and Permanent Teeth with Reversible Pulpitis.

Recommendation:

In primary and permanent teeth with deep caries diagnosed with reversible pulpitis, clinicians should attempt conservative treatment, as the inflamed dental pulp, if not exposed, can heal when provided with a favorable environment. In these cases, the inflammatory process stimulates odontoblasts to produce reactionary dentine. (Consensus-based Recommendation, Strength: Conditional)

Summary of Evidence:

The cellular, neural, and vascular components of dental pulp coordinate inflammation for its protection and initiate repair [1]. Pulpal cells release pro-inflammatory cytokines (TNFα, IL-1β, IL-8, IL-6), often followed by anti-inflammatory mediators (IL-4, IL-10) and growth factors that moderate inflammation and promote healing [1]. Dental nerves contribute through neuropeptides like substance P and CGRP, which mediate neurogenic inflammation. Neuropeptides also exhibit antimicrobial properties against cariogenic bacteria [2] and facilitate healing by releasing growth factors [3]. Recent evidence indicates that in slowly progressing deep carious lesions, low-grade inflammation stimulates odontoblast activity and reactionary dentine formation, protecting the pulp [4 5 6]. Clinical, radiographic, and histological evaluations confirm reactionary dentine formation in deep carious lesions treated with selective caries removal and indirect pulp treatment (IPT) [7 8 9].

2. Pulpal Response to Extremely Deep Caries with Pulp Exposure in Primary and Permanent Teeth

Recommendation:

In primary and permanent teeth with extremely deep caries and iatrogenic pulp exposure, severe injury causes odontoblast cell death and the inflammatory response triggers differentiation of the dental pulp stem cells to produce reparative dentine, if an environment favorable for healing can be created. (Consensus-based Recommendation, Strength: Conditional)

Summary of Evidence:

In extremely deep caries, the lesion extends into dental pulp, causing pathological carious exposure and bacterial invasion triggering severe inflammation and subsequent odontoblast cell death.[10] Signaling molecules produced during the inflammatory process, in conjunction with bioactive

molecules released from damaged dentine, are posited to play an important role in activating and recruiting resident stem cells to the injury site. The dental pulp stem cells will then undergo differentiation into odontoblast- like cells to produce reparative dentine. [11] Although the mechanism of reparative dentine formation is not fully understood, numerous studies validate this process through evaluations of direct pulp capping and partial pulpotomy outcomes, where the formation of dentine barrier has been demonstrated both radiographically and histologically. [12-14]

3. Pulpal Diagnosis in Primary and Permanent Teeth

Recommendation:

Pulpal diagnosis requires a comprehensive assessment and documentation of the patient's medical and dental history, chief complaint, along with the results of diagnostic tests, and clinical and radiographic examinations. Key signs and symptoms to be considered include the onset, course, duration, intensity, aggravating and relieving factors, nocturnal pain, as well as evoked and persistent pain. For permanent teeth, the painful response and duration to cold stimuli should be assessed. For primary teeth, clinicians should primarily rely on clinical signs and symptoms of provoked versus spontaneous pain, along with radiographic findings. (Consensus-based Recommendation, Strength: Conditional)

Summary of Evidence:

Currently, optimal pulp diagnosis combines medical, dental, and chief complaint histories with clinical and radiographic examinations. For permanent teeth, cold and electric pulp testing provides additional diagnostic information. Key assessment factors for VPT include pain duration and pattern (spontaneous/nocturnal vs. provoked), and in permanent teeth, cold response duration (>30 seconds indicates potential issues), pain intensity relative to control teeth, as well as exacerbating and relieving factors. The dental pulp's rich sensory innervation provides reliable evidence of sensibility and sensitivity when activated. [1]

While cold and electric pulp testing show high accuracy in permanent teeth [15], current tests cannot determine inflammation severity or the pulp's healing capacity [16]. Histological studies have revealed that combined coronal and root pulp inflammation is rare, with research showing complete absence of inflammation and bacteria in the radicular pulp of teeth clinically diagnosed with irreversible pulpitis.[17] Studies exploring chairside molecular biomarker tests may eventually help identify pulpal disease markers [18-20], but commercial biomarker tests and thresholds for irreversible disease are not yet available.

Microcirculation assessment measuring oxygen concentration may offer more accurate pulp vitality evaluation. Technologies such as pulse oximetry and laser Doppler flowmetry are under investigation, with ongoing research into their reliability and significance for pulp vitality testing. [21]

The AAPD guidelines recommend VPT for carious primary teeth with normal pulp or reversible pulpitis (provoked pain lasting 5-10 minutes).[22, 23] Teeth presenting with sinus tracts and furcation radiolucency are not suitable for VPT.

4. Diagnostic Accuracy of Pulp Sensibility Testing in Mature, Immature, and Primary Teeth

Recommendation:

In mature permanent teeth, cold testing exhibits high sensitivity, specificity, and accuracy, even in crowned teeth. It can be complemented by electric pulp testing when the results are inconclusive. Given its limited reliability, diagnostic pulp testing should be carried out on a case-by-case basis in primary teeth and immature permanent teeth, using clinical judgement to interpret results. (Consensus-based Recommendation, Strength: Conditional)

Summary of Evidence:

Based on a systematic review, pulp sensibility testing shows high accuracy in mature permanent teeth, with cold testing demonstrating sensitivity of 0.87 and specificity of 0.84, while electric pulp testing (EPT) shows sensitivity of 0.72 and specificity of 0.93. [15] The overall accuracy of cold and electric testing was 82-84%, compared to heat testing at 72%.

Cold testing has traditionally been more reliable than EPT in children and young patients.[24, 25] A recent study showed that both EPT and cold can show high responsiveness in successful cases with partial and full pulpotomy (FP), and that consideration of both tests improves the predictability of a positive outcome.[26] For cold testing, accuracy is higher in teeth without full crowns (92%) than in crowned teeth (87%), though the accuracy in crowned teeth remains impressive.[27]

While pulp sensibility testing is less commonly performed on primary and immature permanent teeth due to children's apprehension and potential difficulty interpreting their responses, research shows these tests may be effective. In primary molars, EPT achieved 89% accuracy, with cold and heat testing reaching 71% and 78% accuracy respectively. [28] Similarly, both cold testing and EPT on intact mature and immature permanent teeth proved accurate with no false responses. [29] Therefore, practitioners should consider pulp testing for primary or immature permanent teeth on a case-by-case basis, and interpret results in context of clinical and radiographic findings. Sensibility testing in traumatized teeth is further discussed in Recommendation 30.

5. Limitations of Current Diagnostic Tools for Assessing Pulpal Inflammation and Healing Potential

Recommendation:

Current diagnostic tests cannot definitively assess the degree of inflammation or the healing potential of the dental pulp in primary and permanent teeth. Although there are no commercially available devices for clinical use, the assessment of the pulp's microcirculation and vitality may be determined by measuring oxygen saturation. Currently, the biomarkers of inflammation have limited utility in clinical practice due to lack of established thresholds. (Consensus-based Recommendation, Strength: Conditional)

Summary of Evidence:

Diagnosing pulp inflammatory status faces significant limitations in current research methodologies. Studies on pulp sensibility testing primarily distinguish between vital and necrotic pulps using direct exposure as the reference standard. This limits the evaluation in cases without the need for root canal treatment, including cases with asymptomatic irreversible pulpitis (approximately 40% of cases progressing to necrosis).[30, 31] Multiple studies found no correlation between clinical symptoms and histological inflammatory status [30, 32, 33], and even when correlations exist[17], successful pulpotomy can be performed.

Despite symptoms traditionally indicating irreversible pulpitis (spontaneous pain, lingering cold response >30 seconds), pulpotomy may succeed in the absence of apical periodontitis. [34-37] Apical periodontitis, however, reduces the success of VPT.[38, 39]

Alternative diagnostic approaches include vascularity assessment via laser Doppler flowmetry and pulse oximetry (97% accuracy)[15], though these lack commercial availability. Direct pulp inspection after exposure is increasingly emphasized to identify necrosis, purulence, or prolonged bleeding[40], with partial VPT sometimes possible in multi-canaled teeth with localized necrosis.[41, 42]

Research on molecular biomarkers aims to improve diagnosis[18-20], but clinical correlation with treatment outcomes remains crucial and poorly studied.[43] While MMP-9 initially showed promise for predicting VPT outcomes[44, 45], long-term results were inconsistent.[46]

6. Pulpal Bleeding Duration as a Diagnostic and Prognostic Indicator in Vital Pulp Therapy

Recommendation:

The duration of pulpal bleeding should be considered alongside other diagnostic indicators of pulpal health rather than in isolation. Although prolonged bleeding has not been definitively established as a predictor of outcomes of VPT, achieving hemostasis may be viewed to be of practical and pragmatic importance for the success of VPT. (Consensus-based Recommendation, Strength: Conditional)

Summary of Evidence:

Despite assumptions that pulpal bleeding and hemostasis time could indicate inflammation severity, scientific evidence supporting this is lacking.

Mutluay et al. (2018)[47] found no correlation between hemostasis time and inflammatory cytokine levels in primary teeth. Recent studies in permanent teeth similarly showed no relationship between bleeding time and pulpal blood inflammatory markers.[48] Clinical outcome studies further demonstrate no association between hemostasis time and VPT success. Linsuwanont et al. (2017)[49] reported that bleeding time poorly differentiated reversible from irreversible pulpitis and did not predict VPT outcomes. Additional studies confirmed that bleeding control time does not influence VPT success.[50, 51]

Recommended hemostasis times vary widely from 1-10 minutes.[52, 53] The ESE currently suggests a 5-minute threshold based on expert opinion. Currently, no evidence links hemostasis time to VPT outcomes.[35] Though hemostasis time

may not predict treatment success, effective bleeding control remains pragmatically important for successful VPT.

7. Use of Interim Therapeutic Restoration (ITR) in Primary Teeth with Deep Caries as a Diagnostic Tool for Pulp Vitality

Recommendation:

In primary teeth with deep caries, clinicians may consider placing a temporary glass ionomer restoration without any caries tissue removal or after selective caries removal to soft dentin (interim therapeutic restoration) for a duration of 1-3 months as a diagnostic tool for pulp vitality before proceeding with definitive treatment. The interim restoration must provide an adequate seal to prevent bacterial ingress while monitoring pulpal response. (Consensus-based Recommendation, Strength: Conditional)

Summary of Evidence:

A retrospective study of 117 primary molars demonstrated improved diagnostic success when interim therapeutic restorations (ITRs) using glass ionomer were placed for 1-3 months before VPT.[54] The success rate of subsequent VPT (pulpotomy or indirect pulp treatment) was significantly higher in teeth receiving ITRs (94%, 50/53) compared to those without ITRs (78%, 50/64) (p=0.01). This improvement was particularly notable in teeth with proximal lesions (p=0.007). Lula et al. (2011)[55] reported reduced bacterial counts and arrested caries progression in primary teeth after partial or selective caries removal followed by sealing for 3-6 months.

WG 1.2 Caries Excavation (Table 3)

8. Use of Magnification for Direct Visualization in Management of Deep and Extremely Deep Carious Lesions

Recommendation:

For the management of deep and extremely deep carious lesions, particularly in cases of pulp exposure, it is suggested to utilize magnification for direct visualization of the pulp, including pulpal bleeding, homogeneity of exposure, and to identify any areas of necrosis and degeneration. (Consensus-based Statement, Strength: not determined)

Summary of Evidence:

The limitations of pulpal diagnostic methods outlined previously and in the absence of molecular diagnostic methods for pulpitis, it has been suggested that direct observation in case of pulp exposure may help determine the status of the dental pulp and aid the clinical decision on the applicability of VPT. The AAE position statement on VPT recommended the use of magnification for direct observation of the exposed pulp to identify areas of degeneration or necrosis.[40] The use of magnification in VPT is also recommended by the recent ESE clinical practice guidelines for endodontic treatment.[35]

9. Conservative Management of Deep Caries Using Selective or Stepwise Caries Removal

Recommendation:

After confirming pulp vitality and considering the stage of tooth development, as well as the ability to place a well-sealed restoration, clinicians should adopt a conservative approach to managing deep caries in both primary and permanent teeth. It is recommended to use selective caries removal or stepwise removal over non-selective (complete) caries removal, except in cases of extremely deep caries. Selective caries removal offers an additional advantage as it is completed in a single visit. (Consensus-based Recommendation, Strength: Strong)

Summary of Evidence:

Selective caries removal to soft dentine (SCRSD) in a single visit offers a lower risk of pulp exposure compared to non-selective (complete) caries removal, while better preserving tooth structure.[56] Maltz et al. (2012a)[57] demonstrated that complete caries removal before restoration is unnecessary, as sealed carious dentine following SCRSD becomes less infected over time compared to dentine left after conventional caries removal.

A multicenter randomized controlled trial by Maltz et al. (2018b)[58] compared single-visit SCRSD with stepwise (SW) excavation over 5 years, concluding that maintaining some carious dentine does not compromise pulp vitality. These findings indicate that reopening the cavity to remove residual infected dentin is unnecessary, as confirmed in both 3-year[59] and 5-year follow-up[58] studies.

A recent systematic review reported that single-session selective caries removal resulted in high success rates and fewer pulpal exposures compared to total removal and two-session (SW) excavation at 18 months follow-up.[60] This is consistent with a recent Cochrane review[61], which found higher failure odds for SW excavation compared to single-session selective removal (OR 2.25, 95% CI 1.33 to 3.82; 3 studies, 371 teeth) with moderate-certainty evidence.

10. Management of Deep Carious Lesions in Vital Primary Teeth Using Hall Technique or Selective Caries Removal

Recommendation:

For vital primary teeth with multi-surface or large single-surface deep carious lesions, either no carious tissue removal with Hall Technique or selective caries removal to soft dentin followed by a preformed crown is recommended over non-selective (complete) caries tissue removal. (Consensus-based Recommendation, Strength: Strong)

Summary of Evidence:

A comprehensive Cochrane review analyzing 17 trials found lower failure rates with the Hall Technique compared to conventional restorations and non-restorative cavity control, with comparable outcomes between the Hall Technique and selective excavation.[61]

Nine recent studies largely confirmed these findings. [62-70] Stepwise and selective caries removal showed equivalent outcomes[65, 71], while the Hall Technique and selective excavation demonstrated fewer failures than complete removal.[63, 70, 72, 73]. Network meta-analysis confirmed the highest probability of failure with complete caries removal versus selective excavation or the Hall

Technique.[61] There is evidence for deeper lesions (ICDAS Grade 5, multi-surface) suggesting that selective removal might outperform sealing alone.[64, 66]

11. Use of Dental Dam Isolation in Treatment of Deep and Extremely Deep Carious Lesions

Recommendation:

Considering the risk of pulp exposures, treatment of deep and extremely deep carious lesions should be performed using a dental dam to isolate the lesion and protect against bacterial contamination. Dental dam isolation is not needed when using Hall technique for management of deep caries in primary teeth. (Consensus-based Statement, Strength: not determined)

Summary of Evidence:

Dental dam isolation is recognized as the gold standard for achieving optimal field isolation in management of deep caries, where pulp vitality preservation is critical.[23, 74] An exception for use of dental dam is Hall technique in primary teeth, which involves cementing preformed metal crowns over carious lesions without caries removal or tooth preparation, making field isolation less critical to its success.

Working Group 2: Managing pulpitis and pulp necrosis in primary teeth *(Table 4)*

12. Use of Calcium Silicate Cements (CSCs) for VPT in Primary Teeth with Pulp Exposure

Recommendation:

For vital primary teeth with deep caries, in the event of pulp exposure, clinicians should utilize CSCs as VPT medicament. These biomaterials promote pulp healing, stimulate the formation of reparative dentin, provide an effective seal, and thereby enhance the long-term success of the treatment. (Consensus-based Recommendation, Strength: Strong)

Summary of Evidence:

Primary teeth with carious pulp exposures are treated with direct pulp capping (DPC) or pulpotomy.[23] For pulp exposures in primary teeth, calcium silicate cements (CSCs) demonstrate superior long-term success and reparative dentine formation potential.[22] The 24-month pulpotomy success rates of 94% for MTA and 90% for Biodentine®, significantly higher than formocresol, ferric sulfate, and calcium hydroxide. Zinc oxide eugenol alone showed particularly poor outcomes (65%). Smail-Faugeron et al. (2018) concluded that for pulpotomies, MTA outperformed FC and FS, all of which exceeded CH performance. The AAPD guideline[23] strongly recommends CSCs for pulpotomies. Given the superior performance of MTA and CSCs in pulpotomies, their use in primary tooth DPC is supported.

13. Preference for Selective Caries Removal (IPT) for VPT in Primary Teeth with Healthy or Reversibly Inflamed Pulp

Recommendation:

When evaluating for VPT, selective caries removal (IPT) should be the preferred approach for primary teeth with healthy pulp or reversible pulpitis due to its high success rate. (Consensus-based Recommendation, Strength: Strong)

Summary of Evidence:

A systematic review of caries removal strategies in primary teeth with deep lesions demonstrated that selective caries removal significantly reduces pulpal exposure compared to complete removal, with no significant difference between stepwise and selective approaches.[75]

Selective caries removal followed by placement of a liner (IPT) demonstrated success rates comparable to pulpotomy using standard medicaments, with high evidence certainty. Among all VPT options, IPT achieved the highest overall success rate (97%) at 24 months.[23] The authors recommend prioritizing indirect pulp treatment with selective caries removal as a cost-effective, biological approach for managing primary teeth with deep caries.

14. Direct Pulp Capping (DPC) in Vital Primary Teeth with Minimal Pulp Exposure

Recommendation:

DPC is a viable treatment option for managing pulp exposures measuring less than 1mm in vital primary teeth with deep caries diagnosed with healthy pulp or reversible pulpitis. However, DPC has a lower success rate compared to pulpotomy. (Consensus-based Recommendation, Strength: Conditional)

Summary of Evidence:

The 24-month overall success rate for DPC in primary teeth was 85.8%[22, 74], which is lower than CSC pulpotomy outcomes during the same period.[23] No significant difference was found in DPC success rates based on medicament selection, though the evidence certainty was very low according to GRADE assessment due to serious inconsistency across two meta-analyses.[22]

15. Full Pulpotomy Using CSCs in Primary Teeth with Deep Caries with Pulp Exposure:

Recommendation:

Clinicians should consider performing FP using CSCs in the event of pulp exposure in primary teeth with deep caries and diagnosed with healthy pulp or reversible pulpitis. (Consensus-based Recommendation, Strength: Strong)

Summary of Evidence:

A systematic review[22] analyzing 12- and 24-month pulpotomy studies, along with additional recent systematic reviews[76-78], confirms that CSCs such as MTA and Biodentine demonstrate the highest clinical and radiographical success rates (>90%) for pulpotomies, with no significant differences between these materials.[79]

Concerns about MTA's high cost, mixing difficulty, and extended setting times were addressed in recent randomized controlled trials demonstrating that most

CSCs (including premixed and fast-setting formulations) achieve high success rates with minimal differences between products.[80-87]

16. Management of Primary Teeth with Irreversible Pulpitis Using CSC Pulpotomy:

Recommendation:

Primary teeth that exhibit signs of irreversible pulpitis, based on a history of spontaneous pain, can be managed successfully with a CSC pulpotomy for 12 months or possibly longer, provided the pulp upon access appears to be vital and there are no signs of necrosis or furcal radiolucency. Achieving hemostasis is considered important for the success of this CSC pulpotomy. (Consensus-based Recommendation, Strength: Conditional)

Summary of Evidence:

A systematic review[88] noted six RCTs with 12–24-month data, and several retrospective studies examining pulpotomy for treating irreversible pulpitis (IP) in primary teeth. These studies defined IP as teeth with spontaneous, nocturnal, or lingering pain without signs of necrosis. Lin concluded that pulpotomy with CSCs achieved 94% success over 12 months regardless of the specific CSC used, suggesting this approach as a viable alternative for primary teeth with IP. Success depends on confirming pulp vitality (evidenced by bleeding) and achieving hemostasis[80, 81, 88-90], supported by longer-term cohort studies.[91]

An earlier clinical study[92] showed pain resolution in most children following pulpotomy with Calcium-Enriched Mixture (CEM, a CSC), with 90% success after one year.

17. Non-Vital Pulp Treatment Option in Primary Teeth without Root Resorption

Recommendation:

Pulpectomy can be considered as a non-vital pulp treatment (NVPT) option for primary teeth without preoperative root resorption diagnosed with irreversible pulpitis or necrotic pulp with or without furcal radiolucency. This procedure can be effectively performed using either rotary or manual instrumentation, along with irrigation solutions such as sodium hypochlorite (1-5%), saline, or chlorhexidine (2%). Consensus-based Recommendation, Strength: Conditional).

Summary of Evidence:

A systematic review showed significantly higher pulpectomy success rates in primary teeth without root resorption (89%) compared to those with resorption (47%) at 12 months, with this difference persisting at 24 months (88% vs. 59%).[93] Long-term follow-up (24-60 months) demonstrated sustained success rates (84-90%) in teeth without preoperative root resorption, though the evidence certainty was rated "very low" according to GRADE.

Regarding instrumentation, rotary techniques were approximately 2 minutes faster than manual methods ($P<0.0001$) and achieved 32% more flush fills, but both showed comparable clinical success rates.[93]

Irrigation solution choice minimally impacts treatment success, with comparable success rates across sodium hypochlorite alone (80%), sodium hypochlorite with saline/distilled water (81%), chlorhexidine (87%), and water/saline (81%).[93]

18. Obturation Material and Technique Selection for Primary Tooth Pulpectomy

Recommendation:

When obturating root canal(s) of a primary tooth undergoing pulpectomy, select either Zinc Oxide/Iodoform/Calcium Hydroxide or Zinc Oxide Eugenol (ZOE), over Calcium Hydroxide/Iodoform as filling materials based on 18-month success rates. To avoid overfilling, utilize pluggers for ZOE and lentulo spirals for calcium hydroxide/Iodoform-based materials. (Consensus-based Recommendation, Strength: Conditional)

Summary of Evidence:

For obturation materials, zinc oxide/iodoform/calcium hydroxide (ZO/iodoform/CH) and zinc oxide eugenol (ZOE) showed higher success than Calcium Hydroxide/ iodoform at 18 months. Fill quality using lentulo spirals, hand pluggers, or syringes demonstrated no significant differences. Both ZOE and ZO/iodoform/CH maintained approximately 90% success rates over time, while Calcium Hydroxide/ iodoform success decreased to 71% or lower after 18 months, guiding material selection despite a "very low" certainty evidence rating.[93]

Regarding overfilling rates, Aminabadi et al. (2020)[94] found significant differences based on technique. For zinc oxide-eugenol (ZOE), hand-held lentulo spirals and pluggers showed the lowest overfilling rates (22.7% and 17% respectively), while calcium hydroxide-based materials demonstrated a 14.7% overfilling rate with hand-held lentulo spirals.

19. Pulp Treatment of Primary Teeth with Root Resorption

Recommendation:

Non-Instrumental Endodontic Treatment (NIET), also referred to as Lesion Sterilization and Tissue Repair (LSTR), may be an alternative to pulpectomy for primary teeth with preoperative root resorption diagnosed with irreversible pulpitis or necrotic pulp knowing it has only shown short-term success and has the potential for antibiotic resistance associated with the procedure. (Consensus-based Recommendation, Strength: Conditional)

Summary of Evidence:

Six systematic or scoping reviews have evaluated this technique. Five reviews [95-99] concluded that NIET/LSTR and pulpectomy yield similar clinical results, suggesting both could be utilized clinically. However, Coll et al. 2020[93] found that when stratified by root resorption, pulpectomy showed non-significantly higher success rates in teeth without root resorption, while NEIT/LSTR demonstrated significantly better outcomes in teeth with root resorption.

Several researchers[100-102] have raised theoretical concerns about promoting antibiotic resistance through slowly dissipating medicaments entering systemic

circulation. While no reports of antibiotic resistance related to LSTR appear in the literature, scientists remain concerned that even minute antibiotic amounts in the environment could contribute to resistance development.[103]

20. Restoration of Primary Teeth Following Pulp Therapy

Recommendation:

Following a pulpotomy, NIET (LSTR), or pulpectomy, clinicians should restore the treated tooth with a preformed crown to protect against reinfection and enhance the tooth's long-term survival. (Consensus-based Recommendation, Strength: Conditional)

Summary of Evidence:

A systematic review and meta-analysis by Gillen et al. (2011)[104] emphasized that optimal root canal treatment outcomes depend on both high-quality endodontic treatment and restoration. Coll et al. (2020)[93] compared 12-month pulpectomy success rates between 15 studies using stainless steel crowns (SSCs) and five using composite or amalgam restorations, finding no significant difference between groups. Coll et al. (2020)[93] reported pulpectomy success rates of 90% for SSCs versus 77% for composite restorations at 24 months.

Adhesive sealing of the pulp chamber represents an established method for preventing intraorifice coronal microleakage[105], with growing evidence supporting adhesive resin restoration of the pulp chamber prior to crown placement to prevent leakage-related complications following root canal treatment.[106]

Working Group 3: Managing pulpitis and pulp necrosis in permanent teeth *(Table 5)*

21. Use of CSCs in Vital Pulp Therapy

Recommendation:

For VPT in permanent teeth with deep caries, clinicians should utilise CSCs. These biomaterials promote pulp healing and stimulate the formation of reparative dentine, thereby enhancing the long-term success of the treatment. Glass ionomer cement may be used in the absence of pulp exposure. Placement of a well-sealed coronal restoration enhances the long-term success of the treatment. (Consensus-based Recommendation, Strength: Strong)

Summary of Evidence:

CSCs have significantly improved the outcomes of procedures that approximate dental pulp and periapical tissues due to their biocompatibility, mineralization induction, antimicrobial effects, bioactive dentin interface, and setting expansion.[107, 108] CSCs are now optimal for VPT.[109]

Research demonstrates CSCs outperform calcium hydroxide in direct pulp capping across observational studies.[110], randomized clinical trials[111], and systematic reviews[112], though different CSC types show no significant outcome differences.[112, 113]

CSCs also surpass calcium hydroxide in partial or complete pulpotomy[114, 115], with significant clinical and radiographic improvements at 12 and 24 months.

No significant differences exist among CSC types for carious exposure[116] or irreversible pulpitis cases.[34, 117]
Older bismuth oxide-containing CSCs may discolor dentin, making newer non-staining formulations such as Biodentine (Septodont) and Neo MTA Plus (Avalon Biomed Inc) preferable for anterior teeth.[118]
For selective caries removal or IPT, a randomized clinical trial comparing CSC with glass ionomer cement showed no significant differences at one[38] or two years[119] postoperatively.

22. Selective Caries Removal (IPT) for Deep Caries

Recommendation:

Selective caries removal (also known as IPT), should be considered as a viable option for managing deep caries, but not extremely deep caries, in permanent teeth diagnosed with healthy or reversible pulpitis. (Consensus-based Recommendation, Strength: Strong)

Summary of Evidence:

Selective caries removal (IPT) shows favorable outcomes in deep caries cases.[58, 120-122] These studies defined deep caries as lesions reaching ⅔ to ¾ dentine depth[61], with a clear radiopaque dentine barrier separating caries from the pulp.[123] If selective removal of carious tissue is not feasible SW can be an option.[121, 124]

Histological evidence demonstrates that caries depth directly correlates with pulpal inflammation. When caries approach within 0.5 mm of the pulp, extensive inflammation, bacterial invasion, and superficial necrosis occur[125-128], regardless of clinical symptoms.[30, 32, 129] While clinicians cannot precisely determine carious lesion depth, cases without radiographic pulp barriers are considered "extremely deep caries," indicating probable bacterial pulp penetration.[130]

For extremely deep caries with reversible pulpitis, randomized trials demonstrate CSC pulpotomy superiority over selective caries removal.[131]

23. CSC DPC for Pulp Exposure in Permanent Teeth with Deep Caries

Recommendation:

DPC is a viable treatment option for cases of pulp exposure while removing deep caries in permanent teeth, particularly when the pulp is diagnosed as healthy or reversibly inflamed. Notably, the long-term success rate of DPC in permanent teeth is higher when calcium silicate cements are used as a medicament. (Consensus-based Recommendation, Strength: Conditional)

Summary of Evidence:

Based on available evidence, CSCs are now the medicament of choice for DPC in cariously exposed permanent teeth.[35, 112] DPC in carious teeth presents significant challenges compared to traumatic exposures due to extensive inflammation and potential infection.[132-134]
An observational study demonstrated successful DPC of extensive carious lesions using CSC, sodium hypochlorite, and magnification.[135] This approach,

later termed "Class II" or "enhanced pulp capping protocol"[10, 134], has shown considerable success.[110, 111, 136]

In extremely deep lesions, an enhanced protocol using sodium hypochlorite lavage showed superior one-year outcomes compared to saline.[46] However, four-year pulp survival rates decreased to 55%, questioning DPC efficacy in extremely deep carious lesions.

24. Managing Pulp Exposure in Permanent Teeth with Extremely Deep Caries

Recommendation:

For the management of pulp exposure in permanent teeth with extremely deep caries and various degrees of pulpitis, clinicians can perform either a PP or FP using CSCs, as this approach has demonstrated a high success rate. If hemostasis cannot be achieved when attempting a partial pulpotomy, further tissue removal may be necessary by deepening the partial pulpotomy or progressing to a full pulpotomy. (Consensus-based Recommendation, Strength: Conditional)

Summary of Evidence:

An umbrella review reported high success rates (88.5-90.6%) for both partial and full pulpotomy regardless of preoperative pulpitis classification[137], challenging the notion that irreversible pulpitis requires RCT.[138]

CSCs used in pulpotomy procedures (ProRoot MTA, calcium-enriched mixture, Biodentine) have demonstrated excellent outcomes[53, 139], outperforming calcium hydroxide in promoting dentine bridge formation and resolving inflammation.[140, 141] A recent systematic review confirms CSCs as preferred biomaterials for pulpotomy in reversible and irreversible pulpitis cases in younger populations.[142] Additionally, pulpotomy with CSCs offers a cost-effective alternative to RCT.[143]

While hemostasis has traditionally guided treatment progression, evidence on optimal duration is conflicting.[53, 144] Recent studies show no significant association between hemostasis duration and long-term success[142], suggesting prolonged bleeding doesn't justify more aggressive tissue removal. A cohort study of 1,257 cases demonstrated comparable outcomes regardless of hemostasis time.[39]

Despite promising outcomes from high-quality studies, the recommendation remains "conditional" due to protocol variability and limited long-term data. Future research must focus on refining diagnostic tools and establishing universal guidelines.

25. Full Pulpotomy (FP) as an Alternative to Root Canal Treatment in Mature Permanent Teeth

Recommendation:

Due to its minimally invasive nature and potential for pulp preservation, FP using CSCs can be an alternative treatment to non-surgical root canal treatment for mature permanent teeth with carious pulp exposure and clinical signs and symptoms of irreversible pulpitis, such as spontaneous or lingering pain, provided that there are no signs and symptoms of pulp necrosis. Achieving

hemostasis may be viewed to be important for the success of VPT. The success rate of this full pulpotomy may be comparable to that of root canal treatment for vital permanent teeth. (Consensus-based Recommendation, Strength: Strong)

Summary of Evidence:

Based on histological findings of teeth with irreversible pulpitis showing localized tissue destruction beneath carious pulp exposures[17], the pulp can be preserved through conservative procedures that remove both the stimulus and infected tissue via partial or full pulpotomy.

Clinical studies have reported favorable pulpotomy outcomes in symptomatic apical pulpitis, with success rates ranging from 78-100%.[145-148] Partial pulpotomy using MTA demonstrated an 85% success rate in adults' permanent teeth over a 2-year follow-up period. Randomized controlled studies have shown comparable outcomes between pulpotomy and root canal therapy in teeth with carious pulp exposures diagnosed with either reversible or irreversible pulpitis.[36, 149, 150]

Achieving hemostasis remains essential in VPT, however, studies indicate bleeding time is not a significant prognostic factor in full pulpotomy, with bleeding times up to 10 minutes associated with high success rates.[145, 151] The exact time needed for hemostasis and the point beyond which the pulp requires further amputation remains inconclusive, though one randomized clinical trial found hemostasis time significantly influenced partial pulpotomy outcomes in irreversible pulpitis cases, with 6 minutes as the proposed cutoff point.[152]

26. Pulpotomy in Immature Permanent Teeth with Irreversible Pulpitis

Recommendation:

For immature permanent teeth with extremely deep caries diagnosed with irreversible pulpitis and there are no signs and symptoms of pulp necrosis, FP using CSCs is the recommended VPT due to its effectiveness in supporting root development and maintaining pulp vitality. (Consensus-based Recommendation, Strength: Strong)

Summary of Evidence:

Performing VPT in young teeth has a particular advantage in that it allows for maturation of the root apex (apexogenesis) and further dentine deposition in the cervical area.

Several clinical trials have evaluated PP and FP outcomes in immature teeth with irreversible and reported success rates from 91–100% over 1-3 years follow up[153-156]. Based on results of clinical trials, both the ESE and AAE position statements state that age is no longer a limiting factor for performing VPT procedures in teeth with carious pulp exposure.[10, 40]

The recommendation for pulpotomy using CSCs in irreversible pulpitis in both mature and immature teeth is strong, based on prospective clinical studies and randomized clinical trials with 1-5 years of follow up.

27. Apexification and Regenerative Endodontic Treatments (RETs) for Immature Necrotic Permanent Teeth

Recommendation:

Both apexification and Regenerative Endodontic Treatments (RETs) are viable options for managing immature, necrotic permanent teeth. The use of CSCs, which are supported by evidence, is preferred over calcium hydroxide (CH) apexification. (Consensus-based Recommendation, Strength: Strong)

Summary of Evidence:

Pulp necrosis in immature permanent teeth creates significant endodontic challenges due to their wide canals, open apices, and thin dentinal walls, which complicate standard treatment approaches.[157, 158] Unlike mature teeth, these cases require specialized techniques addressing both infection elimination and structural reinforcement.[159-162]

Traditional apexification using calcium hydroxide technique has declined due to extended treatment time, inconsistent nature of the barrier formed, and progressive weakening of dentine.[163-165] The apical plug technique using CSC (particularly MTA) represents an improved alternative with favorable outcomes, including survival and success rates of 97.1% and 94.6% respectively. [166-174] However, this approach still doesn't promote continued root maturation.[161]

RET aims to restore pulp vitality and facilitate ongoing root development (Lovelace et al. 2011). Clinical studies show promising results with survival rates of 88-100% and success rates of 75-100%.[168, 175-187]

While both apical plug and RET approaches show promise, RET demonstrates superior outcomes for increasing root length and dentinal wall thickness, though the quality of evidence remains low for both techniques.[35, 39, 168, 188-190]

Working Group 4: Managing pulp pathoses in primary and permanent teeth with traumatic injuries *(Table 6)*

28. Pulpal Healing After Traumatic Dental Injuries

Recommendation:

Traumatic dental injuries affect the dental pulp either directly or indirectly due to infection, inflammation, and damage to the periodontium. Evidence supports that dental pulp tissue can heal after dental trauma; therefore, clinicians should allow time for healing especially in cases of mild injury, immature teeth, and younger patients. **(Consensus-based Recommendation, Strength: Conditional)**

Summary of Evidence:

Dental trauma can cause direct or indirect pulp damage in otherwise healthy pulp tissue, unlike caries-affected teeth. Damaged tooth structure may expose dentine tubules, allowing oral contaminants to reach the pulp.[191] The resulting inflammation can trigger tertiary (reactionary or reparative) dentine secretion by odontoblasts to limit damage. Directly exposed pulp tissue can heal effectively when protected from infection.[192] Successful deposition of dentine and long-term pulp preservation have been demonstrated for exposures less than 4 mm when treated within 9 days.[193]

Luxation injuries can compromise pulp health by disrupting the neurovascular supply entering through the apical foramen. As roots mature and apical

openings constrict, the healing capacity becomes limited. The extent and location of injury at the apex determines the ability of pulp to repair.[194-196]

Pulpal response varies by injury type, with milder injuries more likely to heal with fewer consequences.[196-198] The pulp status may deteriorate over time, with signs of necrosis emerging later, necessitating frequent review of traumatic dental injuries.[199]

Evidence supporting these findings primarily comes from animal experiments[194, 195, 199] and clinical cohorts worldwide[196-198], resulting in lower quality evidence and conditional recommendations. Recent observational studies on avulsions suggest that immature teeth have a low chance of spontaneous revascularization.[200]

29. Standardized Documentation and Radiographic Interpretation of Traumatic Dental Injuries

Recommendation:

Traumatic dental injuries must be documented in standardized manner and validated objective criteria must be used for interpreting the radiographic features for clearer prognostic evaluation. (**Consensus-based Recommendation, Strength: Conditional)**

Summary of Evidence:

All traumatic dental injuries present uniquely and should be documented using standardized methods for both clinical practice and research.[201, 202] Essential information from each trauma event should include written and photographic records with baseline investigations, immediate post-procedure documentation, and periodic follow-ups using standardized reproducible criteria as described in the core outcome set.[202] This documentation approach applies to both primary and permanent teeth.

Classification systems have evolved over time, with the most recent NA0D system now adopted in the International Classification of Diseases.[203] A severity index for recording multiple factors in traumatized teeth was initially proposed by Eden and Baysal, later modified to comprehensively describe severity and injuries to hard dental tissues, periodontium, bone, and soft tissues, emphasizing standardized recording for more accurate diagnosis.[204, 205]

Considerable variability in reporting findings and outcomes within dental trauma literature has reduced data quality. The core outcome set for dental injuries addresses this issue by ensuring treatment planning and prognostic assessment details are consistently documented.[202] These outcomes are incorporated in the IADT Guidelines 2020.[201] While studies proposing and validating these classification, index, and core outcome sets follow best practices in qualitative research, the recommendation strength remains conditional due to study design limitations.

30. Impact of Concomitant Luxation Injuries on Pulp Healing in Crown-Fractured Immature Permanent Teeth

Recommendation:

Clinicians should evaluate for concomitant luxation injuries as these can cause disruption of the vascular supply to the pulp negatively impacting healing in immature permanent teeth with crown fractures. **(Consensus-based Recommendation, Strength: Conditional)**

Summary of Evidence:

Crown fractures show significantly reduced success rates when accompanied by luxation injuries. Pulp necrosis occurred in only 3.2% of teeth with isolated enamel-dentin fractures but increased dramatically to 30.1% in cases involving both concussion and mobility.[206] Robertson et al. (2000)[207] found crown fractures without concomitant luxation injury demonstrated 99% pulp survival, 1% pulp canal obliteration (PCO), and 0% necrosis, whereas those with concomitant luxation injuries showed only 70% pulp survival, 25% necrosis, and 5% PCO.

Concurrent luxation injury and complete root development represent key risk factors for pulp necrosis in crown-fractured teeth. The primary factor affecting pulp healing in crown fractures is disruption of the apical vascular supply due to concomitant luxation injuries. Pulp recovery is more favorable in teeth with immature apices in both dentitions.[207] The significance of combination injuries was further substantiated by Lauridsen et al. in 2012.[197, 198]

31. Assessment of Endodontic Intervention Need in Traumatic Dental Injuries

Recommendation:

Clinicians should use a combination of clinical and radiographic signs and symptoms such as pain, infection, discoloration, mobility, and pathological changes in the tooth or bone to assess the need for endodontic intervention in teeth with traumatic injuries. Discoloration in both dentitions may be transient and thereby not a reliable indicator of necrosis. In addition, responses to pulp sensibility tests (thermal, electric, and occasionally, cavity tests) may assist with diagnosis of necrotic pulp in permanent teeth. For primary teeth, pulp sensibility tests are not indicated. A positive response to sensibility tests shortly after trauma is a good predictor of vitality but lack of response is not always associated with subsequent necrosis, especially luxation injuries. **(Consensus-based Recommendation, Strength: Conditional)**

Summary of Evidence:

The pulpal response following traumatic dental injuries (TDIs) is complex due to the wide variety of injury types and severity.[191, 192] Diagnosis is complicated by the absence of accurate clinical measures of pulp inflammation.[192] Pulp sensibility testing becomes unreliable after TDIs and requires cautious interpretation[208], as lack of response does not predict future recovery or necrosis.[191] Sensibility testing has limited value in children due to potential response difficulties and is rarely used in primary dentition.[209, 210]

Thermal and electric pulp tests assess mainly the Aδ nerve fiber response within the pulp-dentine complex.[192, 211] A positive response indicates functioning nerve fibers but doesn't reflect the pulp's histological status and the likelihood of recovery. This response is frequently lost following TDIs[212] and will return over time. Tooth discoloration from blood product diffusion into tubules

may occur in both dentitions but can be transitory and does not necessarily indicate pulp status.[213, 214] Color normalization can occur within months and should be monitored, especially in primary teeth.[209, 215] In permanent teeth, transient apical breakdown with discoloration and periapical radiolucency can be reversible, and endodontic treatment is not indicated based solely on color change.[214]

Pulp canal obliteration (PCO) commonly follows TDIs, manifesting as dense yellow discoloration. Studies show 60% of teeth developing PCO did not respond to pulp testing at injury time.[216] Increased dentine thickness leads to decreased or negative sensibility responses over time.[217] Determining the need for endodontic intervention requires combining clinical and radiographic signs with pulp sensibility responses.

32. Pulpotomy and Direct Pulp Capping in Complicated Crown Fractures

Recommendation:

PP or FP has a high success rate in complicated crown fractures. DPC has a lower success rate in permanent teeth with complicated crown fractures but may be suitable in select cases or as a temporary treatment in uncooperative children until pulpotomy can be performed. Clinicians should use non-staining CSCs to prevent discoloration. **(Consensus-based Recommendation, Strength: Strong for CSC Pulpotomy; Conditional for direct pulp capping)**

Summary of Evidence:

Pulpotomy demonstrates superior clinical, radiographic, and overall success in teeth with complicated crown fractures[210, 218], with CSCs being the material of choice.[218, 219] Randomized clinical trials have established the superiority of CSCs over calcium hydroxide.[219, 220] Both partial and coronal pulpotomy techniques are equally effective in traumatized teeth, with selection depending on tooth and pulp status.[193, 221]

Intraoperative criteria for pulpotomy in delayed treatment of complicated crown fractures mirror those used in deep caries management, including tissue appearance, bleeding duration, and ability to achieve hemostasis. A deeper pulpotomy may be performed when indicated.[221] Evidence supports greater success and pulp survival in crown fractures without associated luxation injuries.[198, 222] These recommendations apply to both mature and immature permanent teeth. While direct pulp capping shows lower success rates, it may be necessary in select cases or as temporary treatment in uncooperative children before pulpotomy can be performed.[210, 223]

33. Immediate Management and Complications in Traumatic Dental Injuries

Recommendation:

Appropriate immediate management can limit the adverse complications of most traumatic injuries; however, avulsion and severe luxation (intrusive, extrusive, and lateral) injuries are associated with a higher incidence of complications. **(Consensus-based Recommendation, Strength: Conditional)**

Summary of Evidence:

Immediate management is crucial to minimize late complications such as pulp necrosis, tooth resorption, apical periodontitis, and marginal bone loss[224], while enabling accurate diagnosis of combination injuries.[197] Immediate intervention is particularly important for complicated crown and crown-root fractures to preserve pulp vitality, especially in immature permanent teeth. Evidence supports VPT even for mature permanent teeth with crown fractures.[221]

For luxation injuries, immediate repositioning and flexible splinting enhance neurovascular healing at the tooth apex, with significantly higher recovery rates in immature teeth.[189] Recent evidence indicates pulp necrosis does not occur in all mature teeth with severe luxation injuries like lateral luxation,[189] however, due to elevated risk, pulp extirpation remains indicated after two weeks for lateral and extrusive luxation.[224]

In intrusive luxation, literature demonstrates increased success for re-eruption in immature permanent teeth regardless of severity, and for mature teeth with intrusion less than 3mm. All recovering teeth require careful monitoring of pulp status. Mature permanent teeth with intrusion of 3-7mm or greater are preferably managed through surgical extrusion with pulp extirpation within two weeks of repositioning. Pulp survival rates are higher in teeth managed orthodontically, making this the preferred approach for immature teeth with intrusion exceeding 3mm that fail to re-erupt.[224, 225] Evidence shows intruded primary teeth should be monitored for re-eruption without pulpal intervention unless infection is evident.[218, 225]

For avulsed permanent teeth, pulp status largely depends on total extra-alveolar time (including both dry and physiological storage periods).[226] Although the prognosis is low, immature permanent teeth can potentially reestablish neurovascular supply, while mature teeth require pulp extirpation within two weeks after replantation.[227, 228] Though cohort studies support these treatment decisions, the recommendation remains conditional due to evidence quality limitations.

34. Follow-Up and Late Complications of Traumatic Dental Injuries

Recommendation:

Appropriate follow up of traumatic injuries is important due to the risk of late complications such as discoloration, pulp necrosis, arrest of root formation, apical periodontitis, external inflammatory root resorption, external replacement (ankylosis-related) resorption, external invasive resorption, and internal resorption. The incidence and management of these complications differ between primary and permanent teeth. **(Consensus-based Recommendation, Strength: Conditional)**

Summary of Evidence:

Traumatic dental injuries trigger responses in both the pulp-dentin and pulp-periodontal complexes.[229] While pulp tissue can heal, these mechanisms may fail due to the location, nature and extent of injury, root apex maturity, infection, re-injury, and other factors.[222] Late complications can include discoloration, pulp necrosis, arrested root formation, apical periodontitis, various forms of root resorption (external inflammatory, replacement, surface, cervical), ankylosis, and internal resorption.[222, 230, 231]

The incidence of these complications and their correlation with different traumatic dental injuries are affected by treatment delay, emergency care quality, patient age, and root apex status. While the pathophysiology of late complications is similar in primary and permanent teeth, their incidence and management strategies vary significantly.[192, 229, 232]

Our understanding of the etiopathogenesis of late complications comes from animal studies, while occurrence data derives from cohort studies. Various systematic reviews have analyzed these data to identify trends and susceptible conditions, but the level of evidence remains low.

35. Management of Traumatized Immature Permanent Teeth with Pulp Necrosis

Recommendation:

Traumatized immature permanent teeth with pulp necrosis can be managed either by RET or by apical plug using CSC barrier. Both methods have demonstrated comparable clinical, radiographic, and overall success and survival rates. However, RET may be preferred for necrotic teeth in very early stages of root development and for teeth with developmental dental anomalies. **(Consensus-based Recommendation, Strength: Conditional)**

Summary of Evidence:

Immature permanent teeth with pulp necrosis following trauma are managed either through RET or apical plug using CSC.[189] Both approaches demonstrate comparable clinical, radiographic, and overall success and survival rates, though success rates are lower following traumatic dental injuries compared to developmental anomalies. RET may be preferable for teeth with very early root development stages and for developmental dental anomalies.[189, 233-235]

Apical barrier technique using CSCs shows clear superiority over calcium hydroxide-based protocols, supported by randomized clinical trials and systematic reviews, substantiating CSC as the medicament of choice.[170, 174, 189] However, a recent umbrella review found no evidence supporting the superiority of any specific CSCs for apical barrier formation or improved prognosis of RET over apical barrier techniques.[189] Evidence favors RET for its potential to induce root elongation and thickening.[189, 233-235] Clinicians should employ objective methods to assess root apex status and periradicular radiolucency.

36. Management of External Root Resorption

Recommendation:

External inflammatory root resorption can be arrested by careful removal of the causative factors by endodontic debridement, irrigation, and use of intracanal medicament (Calcium Hydroxide, Triple Antibiotic Paste, Double Antibiotic Paste). External replacement resorption is progressive in nature and cannot be stopped or reversed. In permanent teeth, decoronation or root submergence or autotransplantation and extraction are acceptable treatments in suitable patients. **(Consensus-based Recommendation, Strength: Conditional)**

Summary of Evidence:

External inflammatory root resorption occurs when osteoclasts activate in response to periodontal injury.[192, 236] Pulp necrosis and its toxic byproducts play a significant role in this process.[192] While pathophysiology is identical in both primary and permanent teeth, primary teeth undergo physiologic resorption that must be distinguished from inflammatory resorption.[210] Infection also contributes significantly to this process.[237]

Management of inflammatory resorption is predictable, requiring pulp extirpation, disinfection, and placement of intracanal medicaments (calcium hydroxide, triple or double antibiotic paste) to control inflammation and microenvironmental acidity.[222, 226]

In contrast, external replacement resorption has a progressive nature that cannot be stopped or reversed.[222, 236] This results from a shift from osteoclastic to osteoblastic activation, potentially causing tooth ankylosis. In growing individuals, decoronation of permanent teeth with replacement resorption will preserve alveolar dimensions.[238] Root submergence, autotransplantation, and extraction are also acceptable treatments in suitable patients.[201, 239-242]

37. Management of Pulp Canal Obliteration

Recommendation:

Pulp canal obliteration in primary and permanent teeth is a healing response and yellow discoloration is due to the internal layers of dentine formed in response to injury. Clinicians should monitor these teeth without any intervention unless pulp necrosis and apical periodontitis develop. **(Consensus-based Recommendation, Strength: Conditional)**

Summary of Evidence:

Pulp canal obliteration (PCO) represents a favorable pulpal response to trauma through tertiary dentine deposition within the pulp space by odontoblast-like cells. This progressive phenomenon occurs in both primary and permanent teeth, eventually leading to complete obliteration of the pulp chamber and calcification of the root canal.[215, 217, 243] The increased dentine thickness associated with PCO can cause tooth discoloration.

Studies have shown that permanent teeth affected by PCO often retain extremely narrow canals containing pulp tissue that may undergo necrosis later, either spontaneously or following repeat trauma. These cases can develop apical periodontitis requiring intervention through guided endodontic treatment or 3D dynamic navigation protocols.[244, 245] In children, PCO generally reflects a vital pulp with preserved defense mechanism and functional integrity. Associated discoloration can be managed through bleaching or veneering procedures.[215]

38. Non-Surgical Endodontic Treatment in Traumatized Permanent Incisors with Apical Periodontitis or Abscess

Recommendation:

In traumatised permanent incisors, the use of non-surgical endodontic protocols for teeth with symptomatic or asymptomatic apical periodontitis or acute abscess with varying grades of periapical radiolucency is indicated in

cases that fail to respond to RET. **(Consensus-based Recommendation, Strength: Conditional)**

Summary of Evidence:

The diagnosis and management of apical periodontitis and acute abscess in traumatized permanent teeth, both mature and immature, is essential for long-term survival.[189, 236] Established criteria for pulp necrosis assessment must be followed, with objective evaluation of radiographs or cone beam computed tomography.[189]. Two-dimensional evaluation criteria were established by Orstavik et al. (1986)[246], while three-dimensional assessment methods were developed by Estrela et al. (2008)[247]. Baseline recording of lesion size and characteristics enables prognostic assessment during follow-ups.

The fundamental rationale for all endodontic treatment involves debridement, disinfection, and reversal of osteoclastic activity by controlling inflammatory mediators and the acidic microenvironment. This approach has proven effective and should be implemented before considering surgical endodontic procedures.[189]

Reference List

1. El Karim I, Cooper PR. Deciphering Reparative Processes in the Inflamed Dental Pulp. Frontiers in Dental Medicine 2021;2:651219:1-10. https://doi.org/10.3389/fdmed.2021.651219
2. El Karim I, Linden G, Orr D, Lundy F. Antimicrobial activity of neuropeptides against a range of micro-organisms from skin, oral, respiratory and gastrointestinal tract sites. J Neuroimmunol 2008;200(1-2):11-16.
3. El Karim I, Linden G, Irwin C, Lundy F. Neuropeptides Regulate Expression of Angiogenic Growth Factors in Human Dental Pulp Fibroblasts. J Endod 2009;35(6):829-33.
4. Smith A, Cassidy N, Perry H, et al. Reactionary dentinogenesis. Int J Dev Biol 1995;39(1):273-80.
5. Bjørndal L, Darvann T. A light microscopic study of odontoblastic and non-odontoblastic cells involved in tertiary dentinogenesis in well-defined cavitated carious lesions. Caries Res 1999;33(1):50-60.
6. Cooper P, Mclachlan J, Simon S, Graham L, Smith A. Mediators of inflammation and regeneration. Advances of Dental Research 2011;23:290-95.
7. Bjørndal L, Larsen T, Thylstrup A. A clinical and microbiological study of deep carious lesions during stepwise excavation using long treatment intervals. Caries Res 1997;31(6):411-7.
8. Maltz M, Oliveira E, Fontanella V, Carminatti G. Deep caries lesions after incomplete dentine caries removal: 40-month follow-up study. Caries Res 2007;41(6):493-96.
9. Sahin N, Saygili S, Akcay M. Clinical, radiographic, and histological evaluation of three different pulp-capping materials in indirect pulp treatment of primary teeth: a randomized clinical trial. Clin Oral Investig 2021;25(6):3945-55.
10. Duncan HF, Galler K, Tomson P, et al. European Society of Endodontology position statement: Management of deep caries and the exposed pulp. Int Endod J 2019;52(7):923-34.

11. Smith A, Scheven B, Takahashi Y, et al. Dentine as a bioactive extracellular matrix. Archives of Oral Biology 2012;57:109-21.
12. Nair P, Duncan HF, Pitt Ford T, Luder H. Histological, ultrastructural and quantitative investigations on the response of healthy human pulps to experimental capping with mineral trioxide aggregate: a randomized controlled trial. Int Endod J 2008;41(2):128-50.
13. Fransson H, Wolf E, Petersson K. Formation of a hard tissue barrier after experimental pulp capping or partial pulpotomy in humans: an updated systematic review. Int Endod J 2016;49(6):533-42.
14. Didilescu A, Cristache C, Andrei M, Voicu G, Perlea P. The effect of dental pulp-capping materials on hard-tissue barrier formation: A systematic review and meta-analysis. J Am Dent Assoc 2018;149(10):903-17.e4.
15. Mainkar A, Kim S. Diagnostic Accuracy of 5 Dental Pulp Tests: A Systematic Review and Meta-analysis. J Endod 2018;44(5):694-702.
16. Donnermeyer D, Dammaschke T, Lipski M, Schäfer E. Effectiveness of diagnosing pulpitis: A systematic review. Int Endod J 2023;56(Suppl 3):296-325.
17. Ricucci D, Loghin S, Siqueira JJ. Correlation between clinical and histologic pulp diagnoses. J Endod 2014;40(12):1932-39.
18. Rechenberg D, Galicia J, Peters O. Biological Markers for Pulpal Inflammation: A Systematic Review. PLoS One 2016;11(11):e0167289.
19. Zanini M, Meyer E, Simon S. Pulp Inflammation Diagnosis from Clinical to Inflammatory Mediators: A Systematic Review. J Endod 2017;43(7):1033-51.
20. Karrar R, Cushley S, Duncan HF, et al. Molecular biomarkers for objective assessment of symptomatic pulpitis: A systematic review and meta-analysis. Int Endod J 2023;56(10):1160-77.
21. Karayilmaz H, Kirzioglu Z. Comparison of the reliability of laser Doppler flowmetry, pulse oximetry and electric pulp tester in assessing the pulp vitality of human teeth. J Oral Rehabil 2011;38(5):340-7.
22. Coll J, Dhar V, Chen C. Primary tooth vital pulp treatment interventions: Systematic review and meta-analyses. Pediatr Dent 2023;45(6):474-96; E51-E100.
23. Coll J, Dhar V, Chen C. Use of Vital Pulp Therapies in Primary Teeth 2024. Pediatr Dent 2024;46(1):13-26.
24. Fulling H, Andreasen J. Influence of maturation status and tooth type of permanent teeth upon electrometric and thermal pulp testing. Scand J Dent Res 1976;84(5):286-90.
25. Fuss Z, Trowbridge H, Bender I, Rickoff B, Sorin S. Assessment of reliability of electrical and thermal pulp testing agents. J Endod 1986;12(7):301-05.
26. Sangwan P, Ramani A, Mishra S, et al. Pulp sensibility responses following complete and partial pulpotomy in mature permanent molars with carious pulpal exposure and symptomatic irreversible pulpitis: Analysis of pooled data from three randomized clinical trials. Int Endod J 2025;58(3):420-33.
27. Hazard M, Wicker C, Qian F, Williamson A, Teixeira F. Accuracy of cold sensibility testing on teeth with full-coverage restorations: a clinical study. Int Endod J 2021;54(7):1008-15.
28. Hori A, Poureslami H, Parirokh M, Mirzazadeh A, Abbott P. The ability of pulp sensibility tests to evaluate the pulp status in primary teeth. Int J Paediatr Dent 2011;21(6):441-45.

29. Cagirir Dindaroglu F, Ozay Gungor N. Comparison of the vitality test with sensitivity tests in mature and immature teeth: clinical trial. BMC Oral Health 2024;24(1):613.
30. Hasler J, Mitchell D. Painless pulpitis. J Am Dent Assoc 1970;81(3):671-77.
31. Michaelson P, Holland G. Is pulpitis painful? Int Endod J 2002;35(10):829-32.
32. Seltzer S, Bender I, Ziontz M. The dynamics of pulp inflammation: correlations between diagnostic data and actual histologic findings in the pulp. Oral Surg Oral Med Oral Pathol 1963;16:846-71.
33. Dummer P, Hicks R, Huws D. Clinical signs and symptoms in pulp disease. Int Endod J 1980;13(1):27-35.
34. Cushley S, Duncan HF, Lappin M. Pulpotomy for mature carious teeth with symptoms of irreversible pulpitis: A systematic review. J Dent 2019;88:103158.
35. Duncan HF, Kirkevang LL, Peters OA, et al. Treatment of pulpal and apical disease: The European Society of Endodontology (ESE) S3-level clinical practice guideline. Int Endod J 2023;56(Suppl 3):238-95.
36. Taha N, Abuzaid A, Khader Y. A Randomized Controlled Clinical Trial of Pulpotomy versus Root Canal Therapy in Mature Teeth with Irreversible Pulpitis: Outcome, Quality of Life, and Patients' Satisfaction. J Endod 2023;49(6):624-31.e2.
37. El Karim I, Duncan HF, Fouad A, et al. Effectiveness of full Pulpotomy compared with Root canal treatment in managing teeth with signs and symptOms indicative of irreversible pulpitis: a protocol for prospectiVE meta-analysis of individual participant data of linked randomised clinical trials (PROVE). Trials 2023;24(1):807.
38. Hashem D, Mannocci F, Patel S, et al. Clinical and radiographic assessment of the efficacy of calcium silicate indirect pulp capping: a randomized controlled clinical trial. J Dent Res 2015;94(4):562-68.
39. Asgary S, Roghanizadeh L, Eghbal M, et al. Outcomes and predictive factors of vital pulp therapy in a large-scale retrospective cohort study over 10 years. Sci Rep 2024;14(1):2063.
40. AAE. AAE Position Statement on Vital Pulp Therapy. J Endod 2021;47(9):1340-44.
41. Koli B, Chawla A, Logani A, Kumar V, Sharma S. Combination of Nonsurgical Endodontic and Vital Pulp Therapy for Management of Mature Permanent Mandibular Molar Teeth with Symptomatic Irreversible Pulpitis and Apical Periodontitis. J Endod 2021;47(3):374-81.
42. Motoki O, Fergus D, Yusuke T, et al. Partial Pulpotomy to Successfully Treat a Caries-Induced Pulpal Micro-Abscess: A Case Report. Frontiers in Dental Medicine 2021;2.
43. Fouad A. Molecular Characterization of Irreversible Pulpitis: A Protocol Proposal and Preliminary Data. Frontiers in Dental Medicine 2022;3:867414.
44. Sharma R, Kumar V, Logani A, et al. Association between concentration of active MMP-9 in pulpal blood and pulpotomy outcome in permanent mature teeth with irreversible pulpitis - a preliminary study. Int Endod J 2021;54(4):479-89.
45. Ballal N, Duncan HF, Wiedemeier D, et al. MMP-9 Levels and NaOCl Lavage in Randomized Trial on Direct Pulp Capping. J Dent Res 2022;101(4):414-19.
46. Ballal N, Duncan HF, Wiedemeier D, et al. 4-Year Pulp Survival in a Randomized Trial on Direct Pulp Capping. J Endod 2024;50(1):4-9.

47. Mutluay M, Arıkan V, Sarı S, Kısa Ü. Does Achievement of Hemostasis After Pulp Exposure Provide an Accurate Assessment of Pulp Inflammation? Pediatr Dent 2018;40(1):37-42.
48. Sabeti M, Nikghalb K, Pakzad R, Fouad A. Expression of Selected Inflammatory Mediators with Different Clinical Characteristics of Pulpal Inflammation. J Endod 2024;50(3):336-43.
49. Linsuwanont P, Wimonsutthikul K, Pothimoke U, Santiwong B. Treatment Outcomes of Mineral Trioxide Aggregate Pulpotomy in Vital Permanent Teeth with Carious Pulp Exposure: The Retrospective Study. J Endod 2017;43(2):225-30.
50. Tan S, Yu V, Lim K, et al. Long-term pulpal and restorative outcomes of pulpotomy in mature permanent teeth. Journal of Endodontics 2020;46:383-90.
51. Careddu R, Duncan HF. A prospective clinical study investigating the effectiveness of partial pulpotomy after relating preoperative symptoms to a new and established classification of pulpitis. International Endodontic Journal 2021;54:2156-72.
52. Bogen G, Chandler N. Pulp preservation in immature permanent teeth. Endod Topics 2012;23(1):131-52.
53. Zanini M, Hennequin M, Cousson P. Which procedures and materials could be applied for full pulpotomy in permanent mature teeth? A systematic review. Acta Odontol Scand 2019;77(7):541-51.
54. Coll J, Campbell A, Chalmers N. Effects of glass ionomer temporary restorations on pulpal diagnosis and treatment outcomes in primary molars. Pediatr Dent 2013;35(5):416-21.
55. Lula E, Almeida LJ, Alves C, Monteiro-Neto V, Ribeiro C. Partial caries removal in primary teeth: association of clinical parameters with microbiological status. Caries Res 2011;45(3):275-80.
56. Maltz M, Jardim J, Mestrinho H, et al. Partial removal of carious dentin: a systematic review and meta-analysis. J Dent 2018;79:1-8.
57. Maltz M, Henz S, de Oliveira E, Jardim J. Conventional caries removal and sealed caries in permanent teeth: a microbiological evaluation. J Dent 2012;40(9):776-82.
58. Maltz M, Koppe B, Jardim J, et al. Partial caries removal in deep caries lesions: a 5-year multicenter randomized controlled trial. Clin Oral Investig 2018;22(3):1337-43.
59. Maltz M, Garcia R, Jardim J. Randomized Trial of Partial vs. Stepwise Caries Removal: 3-year Follow-up. Journal of Dental Research 2012;91(11):1026-31.
60. Figundio N, Lopes P, Tedesco T, et al. Deep Carious Lesions Management with Stepwise, Selective, or Non-Selective Removal in Permanent Dentition: A Systematic Review of Randomized Clinical Trials. Healthcare (Basel) 2023;11(16):2338.
61. Schwendicke F, Walsh T, Lamont T, et al. Interventions for treating cavitated or dentine carious lesions. Cochrane Database Syst Rev 2021;7(7):CD013039.
62. Ayedun O, Oredugba F, Sote E. Comparison of the treatment outcomes of the conventional stainless steel crown restorations and the hall technique in the treatment of carious primary molars. Nigerian journal of clinical practice 2021;24(4):584-94.
63. Boyd D, Foster Page L, Moffat S, Thomson W. Time to complain about pain: Children's self-reported procedural pain in a randomised control trial of Hall and

conventional stainless steel crown techniques. International journal of Paediatric Dentistry 2023;33(4):382-93.
64. Dos Santos N, Leal S, Gouvea D, et al. Sealing of cavitated occlusal carious lesions in the dentine of deciduous molars: a two-year randomised controlled clinical trial. Clinical oral investigations 2022;26(1):1017-24.
65. Elhennawy K, Finke C, Paris S, et al. Selective vs stepwise removal of deep carious lesions in primary molars: 24 months follow-up from a randomized controlled trial. Clinical oral investigations 2021;25(2):645-52.
66. Gibbison R, Crozier R. Are RMGIC restorations as effective with or without selective caries removal in primary molars? Evidence-based dentistry 2021;22(4):132-3.
67. Kaptan A, Korkmaz E. Evaluation of success of stainless steel crowns placed using the hall technique in children with high caries risk: A randomized clinical trial. Nigerian journal of clinical practice 2021;24(3):425-34.
68. Neves P, Souza S, Casagrande L, et al. A 12-month Follow-Up Study of Pulp Oxygen Saturation in Deciduous Molars After Selective and Nonselective Carious-Tissue Removal: a Randomized Pilot Trial. Pesquisa brasileira em odontopediatria e clinica integrada 2023;23.
69. Liberman J, Franzon R, Guimaraes L, et al. Survival of composite restorations after selective or total caries removal in primary teeth and predictors of failures: a 36-months randomized controlled trial. Journal of dentistry 2020;93:103268.
70. Pascareli-Carlos A, Tedesco T, Calvo A, et al. Survival rate of the Hall technique compared with resin composite restoration in multi-surface cavities in primary teeth: a 1-year randomized clinical trial. Journal of applied oral science : revista FOB 2023;31:e20230048.
71. Orhan A, Oz F, Orhan K. Pulp exposure occurrence and outcomes after 1- or 2-visit indirect pulp therapy vs complete caries removal in primary and permanent molars. Pediatric Dentistry 2010;32(4):347-55.
72. Araujo M, Innes N, Bonifacio C, et al. Atraumatic restorative treatment compared to the Hall Technique for occluso-proximal carious lesions in primary molars; 36-month follow-up of a randomised control trial in a school setting. BMC Oral Health 2020;20(1):318.
73. Chompu-inwai P, Boonsongsawat K, Sastraruji T, et al. Three incomplete caries removal techniques compared over two years in primary molars with asymptomatic deep caries or reversible pulpitis. Pediatric Dentistry 2015;37(5):41-8.
74. Coll J, Seale N, Vargas K, et al. Primary Tooth Vital Pulp Therapy: A Systematic Review and Meta-analysis. Pediatr Dent 2017;39:16-27.
75. Aïem E, Joseph C, Garcia A, Smaïl-Faugeron V, Muller-Bolla M. Caries removal strategies for deep carious lesions in primary teeth: Systematic review. International Journal of Paediatric Dentistry 2020;30(4):392-404.
76. Guo J, Zhang N, Cheng Y. Comparative efficacy of medicaments or techniques for pulpotomy of primary molars: a network meta-analysis. Clinical oral investigations 2023;27(1):91-104.
77. Jasani B, Musale P, Jasani B. Efficacy of Biodentine versus formocresol in pulpotomy of primary teeth: a systematic review and meta-analysis. Quintessence international (Berlin, Germany : 1985) 2022;53(8):698-705.
78. Firoozi P, Salman B, Aslaminabadi N. Clinical and radiographic comparison of Biodentine and Formocresol: an updated meta-analysis with trial sequential analysis. Eur Arch Paediatr Dent 2022;23(6):855-67.

79. Zhang X, Li Q. Comparing MTA and Biodentine for molar pulpotomy: A comprehensive meta-analysis. Current Journal of Sports Science and Medicine 2023;11(2):19-29.
80. Alnassar I, Altinawi M, Rekab M, Alzoubi H, Abdo A. Evaluation of the efficacy of mineral trioxide aggregate and bioceramic putty in primary molar pulpotomy with symptoms of irreversible pulpitis (a randomized-controlled trial). Clin Exp Dent Res 2023;9(2):276-82.
81. Sabbagh S, Bahrololoomi Z, Sarraf Shirazi A. Comparative evaluation of cervical pulpotomy and pulpectomy for primary molars with irreversible pulpitis: a multicentre randomised controlled trial. Eur Arch Paediatr Dent 2024;25(2):255-65.
82. Abdelwahab D, Kabil N, Badran A, Darwish D, Abd El Geleel O. One-year radiographic and clinical performance of bioactive materials in primary molar pulpotomy: A randomized controlled trial. J Dent 2024;143:104864.
83. Joo Y, Lee T, Jeong S, et al. A randomized controlled clinical trial of premixed calcium silicate-based cements for pulpotomy in primary molars. J Dent 2023;137:104684.
84. Yilmaz S, Keles S. Efficacy of orthoMTA, retroMTA and ferric sulphate as pulpotomy agents in primary molars: a randomized clinical trial. Eur Oral Res 2023;57(3):144-50.
85. Manohar S, Bazaz N, Neeraja G, Subramaniam P, Sneharaj N. A comparative evaluation of four regenerative materials for pulpotomy in primary molars: An in vivo study. Dent Res J (Isfahan) 2022;19:102.
86. Wang S, Peng C, Liu H. Pulpotomy of human primary molars with novel bioceramic material. Beijing Da Xue Xue Bao Yi Xue Ban 2022;54(6):1196-201.
87. Acharya S, Gurunathan D. Comparative Evaluation of Success of NeoPutty Mineral Trioxide Aggregate® with Formocresol as Pulpotomy Medicaments in Primary Molars: A Clinical Study. Indian J Dent Res 2024;35(2):170-75.
88. Lin G, Chin Y, Choong R. Treatment Outcomes of Pulpotomy in Primary Teeth with Irreversible Pulpitis: A Systematic Review and Meta-Analysis. Children (Basel) 2024;11(5):574.
89. Wang Y, Feng T, Wang L, Guo T, Huang X. Comparison of clinical outcomes between mineral trioxide aggregate and bioceramic materials in pulpotomy for treating early chronic pulpitis in deciduous teeth. The Journal of clinical pediatric dentistry 2024;48(4):185-90.
90. Eshghi A, Hajiahmadi M, Nikbakht M, Esmaeili M. Comparison of Clinical and Radiographic Success between MTA and Biodentine in Pulpotomy of Primary Mandibular Second Molars with Irreversible Pulpitis: A Randomized Double-Blind Clinical Trial. Int J Dent 2022;2022:6963944.
91. Hu X, Zhao C, Wang L, et al. A retrospective study on iRoot BP Plus full pulpotomy for primary molars with partial irreversible pulpitis. Hua Xi Kou Qiang Yi Xue Za Zhi 2024;42(2):242-48.
92. Memarpour M, Fijan S, Asgary S, Keikhaee M. Calcium-Enriched Mixture Pulpotomy of Primary Molar Teeth with Irreversible Pulpitis. A Clinical Study. Open Dent J 2016;10:43-9.
93. Coll J, Vargas K, Marghalani A, et al. A Systematic Review and Meta-Analysis of Nonvital Pulp Therapy for Primary Teeth. Pediatr Dent 2020;42(4):256-461.
94. Aminabadi N, Asl Aminabadi N, Jamali Z, Shirazi S. Primary tooth pulpectomy overfilling by different placement techniques: A systematic review and meta-analysis. J Dent Res Dent Clin Dent Prospects 2020;14(4):250-61.

95. Agarwal S, Bendgude V, Kakodkar P. Evaluation of Success Rate of Lesion Sterilization and Tissue Repair Compared to Vitapex in Pulpally Involved Primary Teeth: A Systematic Review. J Conserv Dent 2019;22(6):510-15.
96. Duarte M, Pires P, Ferreira D, et al. Is there evidence for the use of lesion sterilization and tissue repair therapy in the endodontic treatment of primary teeth? A systematic review and meta-analyses. Clin Oral Investig 2020;24(9):2959-72.
97. Garrocho-Rangel A, Jalomo-Ávila C, Rosales-Berber M, Pozos-Guillén A. Lesion Sterilization Tissue Repair (LSTR) Approach Of Non-Vital Primary Molars With A Chloramphenicol-Tetracycline-ZOE Antibiotic Paste: A Scoping Review. J Clin Pediatr Dent 2021;45(6):369-75.
98. Kumar N, Brigit B, Annapoorna B, et al. Effect of triple antibiotic paste and calcium hydroxide on the rate of healing of periapical lesions: A systematic review. J Conserv Dent 2021;24(4):307-13.
99. Alrayes N, Almaimouni Y, Tounsi A, et al. The effect of an antibacterial mixture and non-instrumentation endodontic treatment in primary teeth: A systematic review and meta-analyses. Saudi Dent J 2023;35(6):575-88.
100. Achanta A, Reche A, Dakhale R, Bharate R. A Comprehensive Review of Lesion Sterilization and Tissue Repair: An Alternative for Pulpectomy in Deciduous Teeth. Cureus 2023;15(11):e48218.
101. Ahirwar P, Shashikiran N, Sundarraj R, et al. A clinical trial comparing antimicrobial efficacy of "essential oil of Ocimum sanctum" with triple antibiotic paste as an intracanal medicament in primary molars. J Indian Soc Pedod Prev Dent 2018;36:191-7.
102. Jaya A, Praveen P, Anantharaj A, Venkataraghavan K, Prathibha R. In vivo evaluation of lesion sterilization and tissue repair in primary teeth pulp therapy using two antibiotic drug combinations. Journal of Clinicl Pediatric Dentistry 2012;37(2):189-92.
103. Jutkina J, Rutgersson C, Flach C, Joakim Larsson D. An assay for determining minimal concentrations of antibiotics that drive horizontal transfer of resistance. Sci Total Environ 2016;548-549:131-38.
104. Gillen B, Looney S, Gu L, et al. Impact of the quality of coronal restoration versus the quality of root canal fillings on success of root canal treatment: a systematic review and meta-analysis. J Endod 2011;37(7):895-902.
105. Belli S, Zhang Y, Pereira P, Pashley D. Adhesive sealing of the pulp chamber. J Endod 2001;27(8):521-6.
106. Thyvalikakath T, LaPradd M, Siddiqui Z, et al. Root Canal Treatment Survival Analysis in National Dental PBRN Practices. J Dent Res 2022;101(11):1328-34.
107. Kim J, Nosrat A, Fouad A. Interfacial characteristics of Biodentine and MTA with dentine in simulated body fluid. J Dent 2015;43(2):241-47.
108. Pelepenko L, Saavedra F, Antunes T, et al. Physicochemical, antimicrobial, and biological properties of White-MTAFlow. Clin Oral Investig 2021;25(2):663-72.
109. Parirokh M, Torabinejad M, Dummer P. Mineral trioxide aggregate and other bioactive endodontic cements: an updated overview - part I: vital pulp therapy. Int Endod J 2018;51(2):177-205.
110. Hilton T, Ferracane J, Mancl L. Comparison of CaOH with MTA for direct pulp capping: a PBRN randomized clinical trial. J Dent Res 2013;92(7 Suppl):16S-22S.

111. Kundzina R, Stangvaltaite L, Eriksen H, Kerosuo E. Capping carious exposures in adults: a randomized controlled trial investigating mineral trioxide aggregate versus calcium hydroxide. Int Endod J 2017;50(10):924-32.
112. Cushley S, Duncan HF, Lappin M, et al. Efficacy of direct pulp capping for management of cariously exposed pulps in permanent teeth: a systematic review and meta-analysis. Int Endod J 2021;54(4):556-71.
113. Linu S, Lekshmi M, Varunkumar V, Sam Joseph V. Treatment Outcome Following Direct Pulp Capping Using Bioceramic Materials in Mature Permanent Teeth with Carious Exposure: A Pilot Retrospective Study. J Endod 2017;43(10):1635-39.
114. Li Y, Sui B, Dahl C, et al. Pulpotomy for carious pulp exposures in permanent teeth: A systematic review and meta-analysis. J Dent 2019;84:1-8.
115. Taha NA, Khazali MA. Partial Pulpotomy in Mature Permanent Teeth with Clinical Signs Indicative of Irreversible Pulpitis: A Randomized Clinical Trial. J Endod 2017;43(9):1417-21.
116. Wang W, Zeng Q, Li Y, et al. Effectiveness of pulpotomy in managing carious exposure in mature permanent teeth: A systematic review and meta-analysis. J Dent 2024;150:105392.
117. Li Y, Wang W, Zeng Q, et al. Efficacy of pulpotomy in managing irreversible pulpitis in mature permanent teeth: A systematic review and meta-analysis. J Dent 2024;144:104923.
118. Camilleri J. Staining Potential of Neo MTA Plus, MTA Plus, and Biodentine Used for Pulpotomy Procedures. J Endod 2015;41(7):1139-45.
119. Hashem D, Mannocci F, Patel S, et al. Evaluation of the efficacy of calcium silicate vs. glass ionomer cement indirect pulp capping and restoration assessment criteria: a randomised controlled clinical trial-2-year results. Clin Oral Investig 2019;23(4):1931-39.
120. Maltz M, Oliveira E, Fontanella V, Carminatti G. Deep caries lesions after incomplete dentine caries removal: 40-month follow-up study. Caries Res 2007;41(6):493-6.
121. Dhar V, Pilcher L, Fontana M, et al. Evidence-based clinical practice guideline on restorative treatments for caries lesions: A report from the American Dental Association. J Am Dent Assoc 2023;154(7):551-66.
122. Pilcher L, Pahlke S, Urquhart O, et al. Direct materials for restoring caries lesions: Systematic review and meta-analysis-a report of the American Dental Association Council on Scientific Affairs. J Am Dent Assoc 2023;154(2):e1-e98.
123. Shivakumar K, Prasad S, Chandu G. International Caries Detection and Assessment System: A new paradigm in detection of dental caries. J Conserv Dent 2009;12(1):10-16.
124. Ramezanzade S, Bjørndal L, Chen H, Baysan A. Effectiveness of stepwise or selective in comparison to non-selective caries removal in managing deep caries in vital permanent teeth: A systematic review with trial sequential, pairwise and network meta-analyses. Caries Res 2025:1-52.
125. Reeves R, Stanley H. The relationship of bacterial penetration and pulpal pathosis in carious teeth. Oral Surg Oral Med Oral Pathol 1966;22(1):59-65.
126. Langeland K. Tissue response to dental caries. Endod Dent Traumatol 1987;3(4):149-71.
127. Murray P, Smith A, Windsor L, Mjor I. Remaining dentine thickness and human pulp responses. Int Endod J 2003;36(1):33-43.

128. Ricucci D, Siqueira JJ, Li Y, Tay F. Vital pulp therapy: histopathology and histobacteriology-based guidelines to treat teeth with deep caries and pulp exposure. J Dent 2019;86:41-52.
129. Langeland K. Management of the inflamed pulp associated with deep carious lesion. J Endod 1981;7(4):169-81.
130. Demant S, Dabelsteen S, Bjørndal L. A macroscopic and histological analysis of radiographically well-defined deep and extremely deep carious lesions: carious lesion characteristics as indicators of the level of bacterial penetration and pulp response. Int Endod J 2021;54(3):319-30.
131. Chua S, Sim Y, Wang W, Mok B, Yu V. One-year outcome of selective caries removal versus pulpotomy treatment of deep caries: A pilot randomized controlled trial. Int Endod J 2023;56(12):1459-74.
132. Barthel C, Rosenkranz B, Leuenberg A, Roulet J. Pulp capping of carious exposures: treatment outcome after 5 and 10 years: a retrospective study. J Endod 2000;26(9):525-28.
133. Bjørndal L, Fransson H, Bruun G, et al. Randomized Clinical Trials on Deep Carious Lesions: 5-Year Follow-up. J Dent Res 2017;96(7):747-53.
134. Bjørndal L, Fransson H, Simon S. Treatment of vital pulp conditions. In: Bjørndal L, Kirkevang LL, Whitworth J, editors. Textbook of Endodontology. Oxford, UK: Wiley Blackwell; 2018. p. 61-67.
135. Bogen G, Kim J, Bakland L. Direct pulp capping with mineral trioxide aggregate: an observational study. J Am Dent Assoc 2008;139(3):305-15.
136. Marques M, Wesselink P, Shemesh H. Outcome of Direct Pulp Capping with Mineral Trioxide Aggregate: A Prospective Study. J Endod 2015;41(7):1026-31.
137. Lin G, Yew Y, Lee H, et al. Is pulpotomy a promising modality in treating permanent teeth? An umbrella review. Odontology 2022;110(2):393-409.
138. Asgary S, Eghbal M. Challenging the Misnomer of Irreversible Pulpitis and Deliberating the Urgent Need for Reclassification of Pulpal Diseases Based on the Efficacy of Vital Pulp Therapies: An Overview of Systematic Reviews. Iran Endod J 2023;18(4):202-05.
139. Silva E, Pinto K, Belladonna F, et al. Success rate of permanent teeth pulpotomy using bioactive materials: A systematic review and meta-analysis of randomized clinical trials. Int Endod J 2023;56(9):1024-41.
140. Asgary S, Eghbal M, Parirokh M, Ghanavati F, Rahimi H. A comparative study of histologic response to different pulp capping materials and a novel endodontic cement. Oral Surg Oral Med Oral Pathol Oral Radiol Endod 2008;106(4):609-14.
141. Tabarsi B, Parirokh M, Eghbal M, et al. A comparative study of dental pulp response to several pulpotomy agents. Int Endod J 2010;43(7):565-71.
142. Asgary S, Shamszadeh S, Shirvani A. Management strategies for pulpitis in vital permanent teeth in children and adolescents: a systematic review and meta-analysis of randomized clinical trials. Eur Arch Paediatr Dent 2025.
143. Yazdani S, Jadidfard M, Tahani B, et al. Health Technology Assessment of CEM Pulpotomy in Permanent Molars with Irreversible Pulpitis. Iran Endod J 2014;9(1):23-29.
144. Asgary S, Parhizkar A. Importance of 'Time' on 'Haemostasis' in Vital Pulp Therapy - Letter to the Editor. Eur Endod J 2021;6(1):128-29.

145. Asgary S, Eghbal M, Fazlyab M, Baghban A, Ghoddusi J. Five-year results of vital pulp therapy in permanent molars with irreversible pulpitis: a non-inferiority multicenter randomized clinical trial. Clin Oral Investig 2015;19(2):335-41.
146. Taha N, Abdelkhader S. Outcome of full pulpotomy using Biodentine in adult patients with symptoms indicative of irreversible pulpitis. Int Endod J 2018;51(8):819-28.
147. Taha N, Al-Khatib H. 4-Year Follow-up of Full Pulpotomy in Symptomatic Mature Permanent Teeth with Carious Pulp Exposure Using a Stainproof Calcium Silicate-based Material. J Endod 2022;48(1):87-95.
148. Zhu X, Zhang Y, Wang J, et al. Effect of full pulpotomy using a calcium silicate-based bioactive ceramic in adult permanent teeth with symptoms indicative of irreversible pulpitis: A retrospective study. J Am Dent Assoc 2023;154(6):486-94.
149. Galani M, Tewari S, Sangwan P, et al. Comparative Evaluation of Postoperative Pain and Success Rate after Pulpotomy and Root Canal Treatment in Cariously Exposed Mature Permanent Molars: A Randomized Controlled Trial. J Endod 2017;43(12):1953-62.
150. Asgary S, Eghbal M, Shahravan A, et al. Outcomes of root canal therapy or full pulpotomy using two endodontic biomaterials in mature permanent teeth: a randomized controlled trial. Clin Oral Investig 2022;26(3):3287-97.
151. Taha N, Ahmad M, Ghanim A. Assessment of Mineral Trioxide Aggregate pulpotomy in mature permanent teeth with carious exposures. Int Endod J 2017;50(2):117-25.
152. Taha N, Albakri S. Outcome and Prognostic Factors for Partial and Full Pulpotomy in the Management of Spontaneous Symptomatic Pulpitis in Carious Mature Permanent Teeth: A Randomized Clinical Trial. J Endod 2024;50(7):889-98.
153. Qudeimat MA, Alyahya A, Hasan AA. Mineral trioxide aggregate pulpotomy for permanent molars with clinical signs indicative of irreversible pulpitis: a preliminary study. Int Endod J 2017;50(2):126-34.
154. Taha NA, Abdelkhader SZ. Outcome of full pulpotomy using Biodentine in adult patients with symptoms indicative of irreversible pulpitis. Int Endod J 2018;51(8):819-28.
155. Uesrichai N, Nirunsittirat A, Chuveera P, et al. Partial pulpotomy with two bioactive cements in permanent teeth of 6- to 18-year-old patients with signs and symptoms indicative of irreversible pulpitis: a noninferiority randomized controlled trial. Int Endod J 2019;52(6):749-59.
156. Tzanetakis GN, Papanakou S, Koletsi D, Georgopoulou M. Outcome of Partial Pulpotomy in Immature Permanent Teeth with Symptomatic Irreversible Pulpitis: A Prospective Case Series Assessment. J Endod 2023;49(9):1120-28.
157. AAE. Glossary of Endodontic Terms. American Association of Endodontists. Chicago, IL: American Association of Endodontists; 2003.
158. Wikström A, Brundin M, Mohmud A, Anderson M, Tsilingaridis G. Outcomes of apexification in immature traumatised necrotic teeth and risk factors for premature tooth loss: A 20-year longitudinal study. Dent Traumatol 2024;40:658-71.
159. Cvek M. Prognosis of luxated non-vital maxillary incisors treated with calcium hydroxide and filled with gutta-percha. A retrospective clinical study. Endod Dent Traumatol 1992;8:45-55.

160. Hargreaves K, Diogenes A, Teixeira F. Treatment options: biological basis of regenerative endodontic procedures. J Endod 2013;39:S30-S43.
161. Trope M. Treatment of the immature tooth with a non-vital pulp and apical periodontitis. Dent Clin N Am 2010;54:313-24.
162. Tsilingaridis G, Malmgren B, Andreasen J, Malmgren O. Intrusive luxation of 60 permanent incisors: a retrospective study of treatment and outcome. Dent Traumatol 2012;28:416-22.
163. Frank A. Therapy for the Divergent Pulpless Tooth by Continued Apical Formation. JADA 1966;72:87-93.
164. Andreasen J, Farik B, Munksgaard E. Long-term calcium hydroxide as a root canal dressing may increase risk of root fracture. Dent Traumatol 2002;18:134-7.
165. Kahler S, Shetty S, Andreasen F, Kahler B. The Effect of Long-Term Dressing with Calcium Hydroxide on the Fracture Susceptibility of Teeth. J Endod 2018;44:464-69.
166. Lin J, Lu J, Zeng Q, et al. Comparison of mineral trioxide aggregate and calcium hydroxide for apexification of immature permanent teeth: a systematic review and meta-analysis. J Formos Med Assoc 2016;115:523-30.
167. Ree M, Schwartz R. Long-term success of nonvital, immature permanent incisors treated with a mineral trioxide aggregate plug and adhesive restorations: a case series from a private endodontic practice. J Endod 2017;43:1370-7.
168. Torabinejad M, Nosrat A, Verma P, Udochukwu O. Regenerative endodontic treatment or mineral trioxide aggregate apical plug in teeth with necrotic pulps and open apices: a systematic review and meta-analysis. J Endod 2017;43:1806-20.
169. Torabinejad M, Chivian N. Clinical Applications of Mineral Trioxide Aggregate. J Endod 1999;25:197-205.
170. Duggal M, Tong H, Al-Ansary M, et al. Interventions for the Endodontic Management of Non-Vital Traumatised Immature Permanent Anterior Teeth in Children and Adolescents: A Systematic Review of the Evidence and Guidelines of the European Academy of Paediatric Dentistry. European Archives of Paediatric Dentistry 2017;18:139-51.
171. Chueh L, Huang G. Immature teeth with periradicular periodontitis or abscess undergoing apexogenesis: a paradigm shift. J Endod 2006;32:1205-13.
172. Witherspoon E, Small J, Regan J, Nunn M. Retrospective Analysis of Open Apex Teeth Obturated With Mineral Trioxide Aggregate. J Endod 2008;34:1171-76.
173. Mente J, Leo M, Panagidis D, et al. Treatment outcome of mineral trioxide aggregate in open apex teeth. J Endod 2013;39:20-6.
174. El Meligy O, Avery D. Comparison of apexification with mineral trioxide aggregate and calcium hydroxide. Pediatr Dent 2006;28:248-53.
175. Chrepa V, Joon R, Austah O, et al. Clinical Outcomes of Immature Teeth Treated with Regenerative Endodontic Procedures--A San Antonio Study. J Endod 2020;46:1074-84.
176. Glynis A, Foschi F, Kefalou I, Koletsi D, Tzanetakis G. Regenerative Endodontic Procedures for the Treatment of Necrotic Mature Teeth with Apical Periodontitis: A Systematic Review and Meta-Analysis of Randomized Controlled Trials. J Endod 2021;47:873-82.

177. Yoshpe M, Einy S, Ruparel N, Ganatra S, Kaufman A. Treatment of Necrotic Anterior and Posterior Teeth with Regenerative Endodontic Procedures Using PRF as a Scaffold: A Retrospective Study. Applied Sciences 2022;12:6774.
178. Yan H, De Deus G, Kristoffersen I. Regenerative endodontics by cell homing: a review of recent clinical trials. J Endod 2023;49:4-17.
179. Jeeruphan T, Jantarat J, Yanpiset K. Mahidol study 1: comparison of radiographic and survival outcomes of immature teeth treated with either regenerative endodontic or apexification methods: a retrospective study. J Endod 2012;38:1330-6.
180. Alobaid A, Cortes L, Lo J. Radiographic and clinical outcomes of the treatment of immature permanent teeth by revascularization or apexification: a pilot retrospective cohort study. J Endod 2014;40:1063-70.
181. Silujjai J, Linsuwanont P. Treatment outcomes of apexification or revascularization in nonvital immature permanent teeth: a retrospective study. J Endod 2017;43:238-45.
182. Chan E, Desmeules M, Cielecki M. Longitudinal cohort study of regenerative endodontic treatment for immature necrotic permanent teeth. J Endod 2017;43:395-400.
183. Lin J, Zeng Q, Wei X. Regenerative endodontics versus apexification in immature permanent teeth with apical periodontitis: a prospective randomized controlled study. J Endod 2017;43:1821-7.
184. Ulusoy A, Turedi I, Cimen M, Cehreli Z. Evaluation of blood clot, platelet-rich plasma, platelet rich fibrin, and platelet pellet as scaffolds in regenerative endodontic treatment: a prospective randomized trial. J Endod 2019;45:560-6.
185. Cheng J, Yang F, Li J. Treatment outcomes of regenerative endodontic procedures in traumatized immature permanent necrotic teeth: a retrospective study. J Endod 2022;48:1129-36.
186. Theekakul C, Banomyong D, Osiri S, et al. Mahidol Study 2: treatment outcomes and prognostic factors of regenerative endodontic procedures in immature permanent teeth. J Endod 2024;50:1569-78.
187. Maree M, Nabriski O, Yoshpe M, Lin S, Kaufman A. Time-Dependent Morphological Changes in Traumatic Immature Teeth with Necrotic Pulps Following Regenerative Endodontic Treatment: A Retrospective Study. Dental Traumatology 2025;41:47-58.
188. Meschi N, Palma P, Cabanillas-Balsera D. Effectiveness of revitalization in treating apical periodontitis: a systematic review and meta-analysis. Int Endod J 2023;56:510-32.
189. Tewari N, Devi P, Sampath S, et al. Comparative Effectiveness of Regenerative Endodontic Treatment Versus Apexification for Necrotic Immature Permanent Teeth With or Without Apical Periodontitis: An Umbrella Review. Dental Traumatology 2024;0:1-20.
190. Kahler B, Lu J, Taha N. Regenerative endodontic treatment and traumatic dental injuries. Dent Traumatol 2024;40:618-35.
191. Andreasen J, Løvschall H, Ricucci D. Response of oral tissues to trauma. In: Andreasen J, Andreasen FM, Andersson L, editors. Textbook and Color Atlas of Traumatic Dental injuries to the teeth 5th E: Wiley and Sons; 2019. p. 66-124.
192. Galler K, Weber M, Korkmaz Y, Widbiller M, Feuerer M. Inflammatory Response Mechanisms of the Dentine-Pulp Complex and the Periapical Tissues. Int J Mol Sci 2021;22(3):1480.

193. Cvek M. A clinical report on partial pulpotomy and capping with calcium hydroxide in permanent incisors with complicated crown fracture. J Endod 1978;4(8):232-37.
194. Skoglund A, Tronstad L. Pulpal changes in replanted and auto-transplanted immature teeth of dogs. J Endod 1981;7:309-16.
195. Skoglund A, Tronstad L, Wallenius K. A microangiographic study of vascular changes in replanted and auto transplanted teeth of young dogs. Oral Surg Oral Med Oral Pathol 1978;45(1):17-28.
196. Robertson A. Pulp survival and hard tissue formation subsequent to dental trauma. A clinical and histological study of uncomplicated crown fractures and luxation injuries [SwedDent J; 1997.
197. Lauridsen E, Hermann N, Gerds T, et al. Combination injuries 1. The risk of pulp necrosis in permanent teeth with concussion injuries and concomitant crown fractures. Dent Traumatol 2012;28:364-70.
198. Lauridsen E, Hermann N, Gerds T, et al. Combination injuries 2. The risk of pulp necrosis in permanent teeth with subluxation injuries and concomitant crown fractures. Dent Traumatol 2012;28:371-8.
199. Heide S, Mjor I. Pulp reactions to experimental exposures in young monkey teeth. Int Endod J 1983;16:11-19.
200. Amaro R, Dos Santos L, Lima T, et al. Pulp healing in immature replanted permanent teeth: A competing risk analysis. Dent Traumatol 2021;37(3):447-56.
201. Levin L, Day P, Hicks L. International Association of Dental Traumatology guidelines for the management of traumatic dental injuries: General introduction. Dent Traumatol 2020;36(4):309-13.
202. Kenny K, Day P, Sharif M. What are the important outcomes in traumatic dental injuries? An international approach to the development of a core outcome set. Dent Traumatol 2018;34(1):4-11.
203. Petti S, Andreasen J, Glendor U, Andersson L. NA0D - The new Traumatic Dental Injury classification of the World Health Organization. Dent Traumatol 2022;38(3):170-74.
204. Eden E, Baysal M, Andersson L. Eden Baysal Dental Trauma Index: Face and content validation. Dent Traumatol 2020;36(2):117-23.
205. Eden E, Onetto J, O'Connell A. Extension of a novel diagnostic index to include soft tissue injuries: Modified Eden Baysal Dental Trauma Index. Dent Traumatol 2021;37(6):749-57.
206. Ravn J. Follow-up study of permanent incisors with complicated crown fractures after acute trauma. Scand J Dent Res 1982;90(5):363-72.
207. Robertson A, Andreasen F, Andreasen J, Noren J. Long-term prognosis of crown-fractured permanent incisors. The effect of stage of root development and associated luxation injury. Int J Paediatr Dent 2000;10(3):191-99.
208. Ghouth N, Duggal M, BaniHani A, Nazzal H. The diagnostic accuracy of laser Doppler flowmetry in assessing pulp blood flow in permanent teeth: A systematic review. Dent Traumatol 2018;34(5):311-19.
209. Holan G. Pulp aspects of traumatic dental injuries in primary incisors: Dark coronal discoloration. Dent Traumatol 2019;35(6):309-11.
210. O'Connell A. Contemporary approach for traumatic dental injuries in the primary dentition. Dental Traumatol 2025;41(Suppl1):17-26.

211. Gopikrishna V, Tinagupta K, Kandaswamy D. Comparison of electrical, thermal, and pulse oximetry methods for assessing pulp vitality in recently traumatized teeth. J Endod 2007;33:531-5.
212. Levin L. Pulp and periradicular testing. J Endod 2013;39(3 Suppl):S13-19.
213. Casula L, Cotti E, Capparè P, Musu D. Transient discoloration and apical breakdown after trauma in permanent dentition. J Biol Regul Homeost Agents 2019;33(5):1647-50.
214. Kahler B. Present status and future directions - Managing discoloured teeth. Int Endod J 2022;55(Suppl 4):922-50.
215. Cardoso M, de Carvalho Rocha M. Association of crown discoloration and pulp status in traumatized primary teeth. Dent Traumatol 2010;26(5):413-16.
216. Andreasen F, Zhijie Y, Thomsen B, Andersen P. Occurrence of pulp canal obliteration after luxation injuries in the permanent dentition. Endod Dent Traumatol 1987;3(3):103-15.
217. Bastos J, Cortes M. Pulp canal obliteration after traumatic injuries in permanent teeth-scientifc fact or fiction? Braz Oral Res 2018;32(suppl1):e75.
218. Day P, Flores M, O'Connell A, et al. International Association of Dental Traumatology guidelines for the management of traumatic dental injuries: 3. Injuries in the primary dentition. Dent Traumatol 2020;36(4):343-59.
219. Tewari N, Goel S, Mathur V. Success of medicaments and techniques for pulpotomy of primary teeth: An overview of systematic reviews. Int J Paediatr Dent 2022;32(6):828-42.
220. Abuelniel G, Duggal M, Kabel N. A comparison of MTA and Biodentine as medicaments for pulpotomy in traumatized anterior immature permanent teeth: A randomized clinical trial. Dent Traumatol 2020;36(4):400-10.
221. Matoug-Elwerfelli M, ElSheshtawy A, Duggal M, Tong H, Nazzal H. Vital pulp treatment for traumatized permanent teeth: A systematic review. Int Endod J 2022;55(6):613-29.
222. Yu C, Abbott P. Responses of the pulp, periradicular and soft tissues following trauma to the permanent teeth. Aust Dent J 2016;61(Suppl 1):39-58.
223. Boutsiouki C, Frankenberger R, Krämer N. Relative effectiveness of direct and indirect pulp capping in the primary dentition. Eur Arch Paediatr Dent 2018;19(5):297-309.
224. Bourguignon C, Cohenca N, Lauridsen E. International Association of Dental Traumatology guidelines for the management of traumatic dental injuries: 1. Fractures and luxations. Dent Traumatol 2020;36(4):314-30.
225. Costa L, Ribeiro C, Cantanhede L, et al. Treatments for intrusive luxation in permanent teeth: a systematic review and meta-analysis. Int J Oral Maxillofac Surg 2017;46(2):214-29.
226. Tewari N, Garima J, O'Connell A. Appropriate Terminology for the Time Elapsed From Avulsion of a Permanent Tooth to Replantation: A Scoping Review and Delphi Consensus. Dent Traumatol 2025;41(1):13-28.
227. Fouad A, Abbott P, Tsilingaridis G. International Association of Dental Traumatology guidelines for the management of traumatic dental injuries: 2. Avulsion of permanent teeth. Dent Traumatol 2020;36(4):331-42.
228. Lauridsen E, Andreasen J, Bouaziz O, Andersson L. Risk of ankylosis of 400 avulsed and replanted human teeth in relation to length of dry storage: A re-evaluation of a long-term clinical study. Dent Traumatol 2020;36(2):108-16.

229. Tewari N, Rajeswary A, Wikström A, Tsilingaridis G. Non-Surgical Endodontic Management of Large Periapical Lesions After Traumatic Dental Injuries. Dent Traumatol 2025;41(Suppl 1):43-52.
230. Kallel I, Douki N, Amaidi S, Ben Amor F. The Incidence of Complications of Dental Trauma and Associated Factors: A Retrospective Study. Int J Dent 2020;2020:2968174.
231. Lin S, Pilosof N, Karawani M, et al. Occurrence and timing of complications following traumatic dental injuries: A retrospective study in a dental trauma department. J Clin Exp Dent 2016;8(4):e429-e36.
232. Moccelini B, Santos P, Barasuol J, et al. Prevalence of sequelae after traumatic dental injuries to anterior primary teeth: A systematic review and meta-analysis. Dent Traumatol 2022;38(4):286-98.
233. Wikström A, Brundin M, Lopes M, El Sayed M, Tsilingaridis G. What is the best long-term treatment modality for immature permanent teeth with pulp necrosis and apical periodontitis? Eur Arch Paediatr Dent 2021;22(3):311-40.
234. Nicoloso G, Goldenfum G, Pizzol T, et al. Pulp Revascularization or Apexification for the Treatment of Immature Necrotic Permanent Teeth: Systematic Review and Meta-Analysis. J Clin Pediatr Dent 2019;43(5):305-13.
235. Antunes L, Salles A, Gomes C, et al. The effectiveness of pulp revascularization in root formation of necrotic immature permanent teeth: A systematic review. Acta Odontol Scand 2016;74(3):161-9.
236. Krastl G, Krug R, Nolte D, Kielbassa A. Traumatized teeth: clinical practice guideline for the interim management of teeth with various poor prognosis scenarios in growing patients. Quintessence Int 2022;53(8):722-31.
237. Fouad A. Microbiological aspects of traumatic injuries. Dent Traumatol 2019;35(6):324-32.
238. Malmgren B. Decoronation: how, why, and when? J Calif Dent Assoc 2000;28(11):846-54.
239. Einy S, Kridin K, Kaufman A, Cohenca N. Immediate post-operative rehabilitation after decoronation. A systematic review. Dent Traumatol 2020;36(2):141-50.
240. Mourão C, Resende R. Dental autotransplantation: a viable solution for preserving oral health and function. Evid Based Dent 2024;25(2):77-78.
241. Jaber M, Prasad P, Akeil M, et al. Long-Term Evaluation of Tooth Transplantation: An Umbrella Review. J Clin Med 2024;13(11):3341.
242. Zhang J, Han Y, Zhong H. Accuracy assessment between computer-guided surgery planning and actual tooth position during tooth autotransplantation. Dent Traumatol 2024;40(6):649-57.
243. Abreu M, Fernandes T, Antunes L, Antunes L, Faria L. Prevalence of pulp canal obliteration after traumatic dental injuries: a systematic review and meta-analysis. Braz Oral Res 2024;38:e092.
244. Krastl G, Zehnder M, Connert T, Weiger R, Kühl S. Guided Endodontics: a novel treatment approach for teeth with pulp canal calcification and apical pathology. Dent Traumatol 2016;32(3):240-46.
245. Sharma S, Haldar P, Kumar V, Chawla A, Logani A. Learning Curve for Dynamic Navigation Procedure during Endodontic Management of Permanent Maxillary Anterior Teeth with Pulp Canal Calcification: A Risk-Adjusted Cumulative Summation Analysis of a Single Operator's Experience. J Endod 2025;51(3):295-302.

246. Orstavik D, Kerekes K, Eriksen H. The periapical index: a scoring system for radiographic assessment of apical periodontitis. Endod Dent Traumatol 1986;2(1):20-34.
247. Estrela C, Bueno M, Azevedo B, Azevedo J, Pécora J. A new periapical index based on cone beam computed tomography. J Endod 2008;34(11):1325-31.
